# Disruption of intestinal barrier and immune homeostasis links gut microbiota dysbiosis to aggravated experimental autoimmune myasthenia gravis

**DOI:** 10.3389/fcimb.2026.1726788

**Published:** 2026-04-10

**Authors:** Wenqi Tian, Shanshan Peng, Fanfan Xu, Xiaotong Kong, Ying Li, Wei Zhang, Zhaojun Liu, Huixue Zhang, Jianjian Wang, Lihua Wang

**Affiliations:** Department of Neurology, The Second Affiliated Hospital of Harbin Medical University, Harbin, China

**Keywords:** experimental autoimmune myasthenia gravis(EAMG), fecal microbiota transplantation (FMT), gut microbiota, intestinal barrier, Th17/Treg cell

## Abstract

**Introduction:**

Myasthenia Gravis (MG) is an acquired autoimmune disease. The imbalance between Th17 and Treg cells plays a crucial role in the pathogenesis of MG. A stable intestinal microbiota is essential for maintaining immune balance, a function primarily mediated by the Th17/Treg axis. This study aims to explore the role of gut microbiota in the pathogenesis of experimental autoimmune myasthenia gravis (EAMG) to identify potential new treatment strategies.

**Methods:**

An EAMG model was established in Lewis rats by immunization with the AChRα97-116 peptide segment. The composition and structure of the gut microbiota were analyzed by 16S rRNA sequencing. The serum levels of inflammatory cytokines (IFN-γ, TNF-α, IL-17, IL-10), AChR-Ab, and LPS were measured using the ELISA method. Colon tissues were subjected to Hematoxylin and eosin (H&E) staining, and Claudin-1 and Muc2 expression was analyzed via immunofluorescence. A microbiota disorder animal model was established by administering an antibiotic mixture via gavage, followed by fecal microbiota transplantation. Splenic CD3+CD4+IL-17A+ Th17 cells and CD3+CD4+CD25+Foxp3+ Treg cells were quantified by flow cytometry.

**Results:**

1. Compositional and structural changes of the gut microbiota in EAMG. Compared with HC, the serum levels of IFN-γ, TNF-α, IL-17, and AChR-Ab in EAMG were significantly increased (P < 0.0001), while the serum level of IL-10 was significantly decreased (P < 0.0001), and the serum level of LPS was increased (P < 0.01). The protein levels of Claudin-1 (P < 0.001) and Muc2 (P < 0.05) in the colon were significantly reduced in EAMG. 2. Relative to rats receiving HC microbiota transplantation (HMb), those receiving EAMG microbiota transplantation (MMb) exhibited significantly elevated serum levels of AChR-Ab (P < 0.01), TNF-α (P < 0.05), IL-17 (P < 0.05), and LPS (P < 0.01), alongside significantly reduced colonic levels of Claudin-1 (P < 0.05) and Muc2 (P < 0.0001). 3. Compared with the EAMG group, the ABX + EAMG group (EAMG with microbiota dysbiosis) exhibited significantly lower colonic levels of Claudin-1 and Muc2 (P < 0.05), a significantly greater splenic Th17/Treg cell imbalance (P < 0.01), and significantly higher serum AChR-Ab levels (P < 0.01).

**Discussion:**

The gut microbiota is intricately linked to the progression of EAMG. Microbiota dysbiosis can exacerbate intestinal barrier damage in EAMG and may further influence the pathological changes of myasthenia by disrupting the Th17/Treg immune balance. These findings suggest a novel therapeutic strategy for the treatment of myasthenia gravis by re-establishing microbial homeostasis.

## Graphical Abstract

**Figure:**
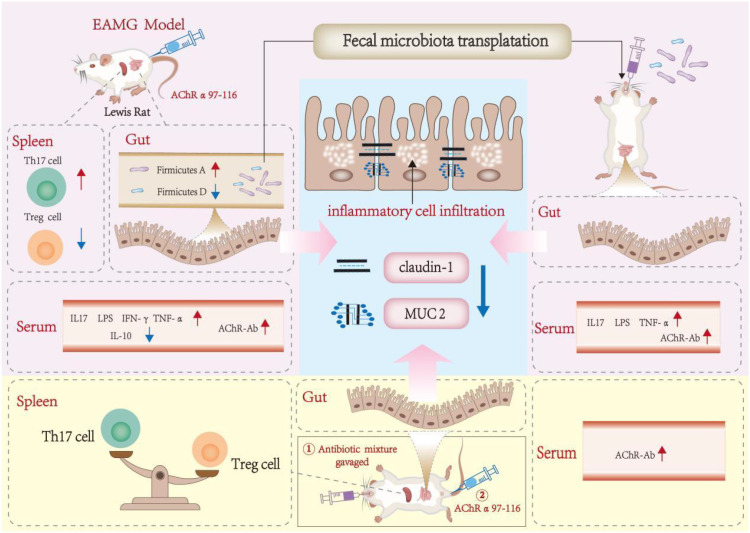
The figure summarizes findings from the experimental autoimmune myasthenia gravis (EAMG) model in Lewis rats. The figure illustrates alterations in the intestinal microbiota, differences in serum cytokine levels, and shifts in immune cells in the spleen. Furthermore, the data demonstrate the effects of fecal microbiota transplantation and dysbiosis induced by antibiotic mixtures on the infiltration of intestinal inflammatory cells, intestinal barrier integrity as assessed by Claudin-1 and Muc2 expression, and the immune response.

## Highlights

- The composition and structure of the gut microbiota in EAMG has changed.
- The gut microbiota of EAMG can damage intestinal barrier function and exacerbate immune homeostasis.
- Microbiota dysbiosis can promote the imbalance of Th17/Treg and aggravate the pathological progression of EAMG.

## 1 Introduction

Myasthenia gravis is a chronic autoimmune disorder that impairs neuromuscular transmission and necessitates lifelong management. Current therapies are constrained by substantial side effects and high costs, driving the search for more effective and safer options.

The antagonism between anti-inflammatory regulatory T (Treg) cells and pro-inflammatory T helper 17 (Th17) cells plays a crucial role in maintaining immune homeostasis (Omenetti and Pizarro, 2015; Zhang et al., 2021). Furthermore, a balanced gut microbiota is equally essential for immune system homeostasis. Numerous studies have demonstrated that gut microbiota modulates the Th17/Treg cell balance, thereby improving various metabolic disorders and autoimmune diseases (Komatsu et al., 2014; Onyekwere et al., 2015). Studies have shown that an imbalance between Th17 and Treg cells can promote the occurrence and development of MG (Aguilo-Seara et al., 2017; Chen and Tang, 2021; Lu et al., 2023). The role of gut microbiota in myasthenia gravis and its relationship to the Th17/Treg balance need to be further explored.

Recent research on gut microbes shows that the diversity and composition of gut microbiota in MG patients differ significantly from those in healthy individuals (Ding et al., 2023). Furthermore, distinct variations are observed in the gut microbiota and metabolite profiles among different MG subtypes (Tan et al., 2020). The results of previous studies suggest that MG alters gut microbiota, and that transplanting human gut microbiota into animals can influence disease symptoms. Zheng et al. demonstrated that transplanting gut microbiota from MG patients into germ-free mice caused observable changes in motor ability. In contrast, germ-free mice that received feces from healthy individuals showed normal motor ability. These findings indicate a correlation between gut microbiota and disease manifestations (Manichanh et al., 2010; Zheng et al., 2019). Genetic factors influence the phenotypic effects of fecal microbiota transplantation, and its efficacy varies among species (Wos-Oxley et al., 2012). Notably, few studies have examined microbiota transplantation within the same genus in myasthenia gravis. Whether changes in gut microbiota are merely a secondary effect of MG pathology or directly contribute to disease pathogenesis remains to be explored. Thus, this study aims to investigate the effect of microbiota transplantation on MG within the same species to minimize confounding factors related to genetic background.

We investigated changes in gut microbiota and intestinal barrier function in experimental autoimmune myasthenia gravis (EAMG). Moreover, we examined how gut microbiota affects the intestinal barrier and clinical manifestations in Lewis rats to elucidate their interactions. Additionally, we assessed the impact of dysbiosis on EAMG symptoms and on the Th17/Treg immune balance. These findings offer new insights into the mechanisms by which gut microbiota influence MG and suggest potential therapeutic strategies for its treatment.

## 2 Materials and methods

### 2.1 Experimental animals

Female Lewis rats aged 6 to 8 weeks, were purchased from Vital River Laboratory Animal Technology Co., Ltd. (Beijing, China). The rats were raised in an SPF-grade animal laboratory of the Second Affiliated Hospital of Harbin Medical University at 22 ± 2°C, with a 12-hour light/dark cycle. The study protocol was approved by the Ethics Committee of the Second Affiliated Hospital of Harbin Medical University (approval number YJSKY2023-119).

### 2.2 Enzyme-linked immunosorbent assay

According to the instructions, levels of Acetylcholine receptors antibody (AChR-Ab) (AD1392Ra, Andygene, Beijing, China), Interleukin-10 (IL-10) (AD3254Ra, Andygene, Beijing, China), Interleukin-17 (IL-17) (AD2572Ra, Andygene, Beijing, China), Interferon-γ (IFN-γ) (AD2593Ra, Andygene, Beijing, China), Tumor necrosis factor-α (TNF-α) (AD3238Ra, Andygene, Beijing, China) and Lipopolysaccharides (LPS) (JONLNBIO, Shanghai, China) were measured by cytokine antibody Elisa kits (**Table 1**).

**Table 1:** Summarizes information regarding the cytokine antibody Elisa kits, including the sensitivity and inspection range.

Elisa kits	Sensitivity	Inspection range
AChR-Ab	15 pg/mL	100 pg/ml - 2300pg/ml
IL-10	0.1 ng/L	2.5 ng/L -50ng/L
IL-17	0.5 pg/mL	5 pg/mL -100 pg/mL
IFN-γ	10 ng/L	100 ng/L -2000ng/L
TNF-α	10 ng/L	15 ng/L -300ng/L
LPS	0.075 ng/L	0.156–10 ng/mL

### 2.3 Establishment of the experimental autoimmune myasthenia gravis (EAMG) model

Initial immunization: EAMG group: Each Lewis rat was injected subcutaneously at the tail root with 200 μL of a mixed emulsion, which contained 50 μg AChRα97–116 peptide (DGDFAIVKFTKVLLDYTGHI, synthesized by Chinapeptides. Co., Ltd., Shanghai, China), 2 mg *Mycobacterium tuberculosis* (TB, H37Ra, BD DIFCO, USA, 231141), equal volume phosphate-buffered saline (PBS; Gibco, Grand Island, NY, USA) and complete Freund’s adjuvant (CFA)(Sigma-Aldrich). Control group: Rats were subcutaneously injected 200 μl of a mixed emulsion containing 1 mg dry powder of *Mycobacterium tuberculosis* equal volume PBS and CFA.

Boost immunization: EAMG rats were injected subcutaneously at the tail root with 200 μL of a mixed emulsion containing 50 μg AChRα97–116 peptide, an equal volume of PBS, and incomplete Freund’s adjuvant (IFA) (Sigma-Aldrich). The control group received a mixed emulsion of 200 μL, consisting of an equal volume of PBS and IFA.

#### 2.3.1 Lennon criteria

The observer holds the end of the rat’s tail so that it is held upside down, then drags it backward and upward until the rat releases its grip on the grid. The observer repeats this operation for 30 seconds and observes the rat’s behavior (**Table 2**) (Lennon et al., 1975).

**Table 2:** Summarizes the Lennon clinical symptom grading criteria.

Lennon criteria	Clinical symptom
0 points	Normal muscle strength
1 point	Slight weakening in activity, gripping, or braking; if symptoms significantly worsen after 30 seconds of exercise, it is considered a positive fatigue test
2 points	Clinical signs before exercise, including tremor, bowed head, bowed back, and weak grip strength
3 points	Severe clinical symptoms, near death
4 points	Death

Symptoms that fall between two integer scores are assigned an intermediate score of 0.5 points.

### 2.4 Study design

Section 1 Lewis rats were divided into two groups: the HC group and the EAMG group, with six rats in each group. We defined Day 0 as the day of initial immunization according to the established EAMG model procedure. Weight measurements and behavioral scoring were performed every other day throughout the experiment. Thereafter, on Day 30, a booster dose was administered according to the established EAMG protocol. Behavioral scoring followed the Lennon criteria, which assess clinical symptoms of experimental autoimmune myasthenia gravis. The experiment ended when the EAMG group showed significant weight loss alongside a continuous increase in behavioral scores. We measured serum levels of acetylcholine receptor antibodies (AChR-Ab), interleukin-10 (IL-10), interleukin-17 (IL-17), interferon-gamma (IFN-γ), tumor necrosis factor-alpha (TNF-α), and lipopolysaccharide (LPS) using cytokine antibody ELISA kits. Intestinal tissues were stained with Hematoxylin and Eosin staining (HE) and subjected to immunofluorescence targeting Claudin-1 and Muc2 in the colon to assess tissue structure and barrier function. Finally, 16S rRNA gene sequencing analysis was performed to compare gut microbiota diversity between the two groups.

Section 2 We conducted a fecal microbiota transplantation experiment using two sets of fecal samples obtained in the initial step. The aim was to investigate whether the gut microbiota could induce pathological changes in rats. Firstly, we administered an antibiotic mixture by gavage daily for 14 days, starting from day 0. After 14 days of intragastric administration, fecal samples from both groups were collected for 16S rRNA sequencing. Failure of library construction was considered an indicator of effective microbiota depletion before transplantation. The next step, fecal microbiota transplantation, then began using microbiota originating from the HC and EAMG groups in Section 1. The transplantation was performed daily for 7 days. At the end of the experiment, fecal samples from both groups were collected for 16S rRNA gene sequencing analysis. We measured serum levels of AChR-Ab, IL-10, IL-17, IFN-γ, TNF-α, and LPS using cytokine antibody ELISA kits. We also performed HE staining and immunofluorescence staining targeting colon Claudin-1 and Muc2 to evaluate intestinal tissue structure and barrier function. Comparison of microbial communities between donors and recipients confirmed successful colonization and representativeness of the transplanted microbiota.

Section 3 The gut microbiota and EAMG are mutually reinforcing. To further investigate the impact of gut microbiota disorder on EAMG, we established a microbiota dysbiosis rat model (refer to “Establish the animal model of microbiota dysbiosis”). The Lewis rats were divided into three groups: HC group, EAMG group and antibiotic (ABX) + EAMG group. The ABX+ EAMG group was gavaged with the antibiotic mixture, while the HC group and EAMG group were gavaged with equal volumes of PBS. After 14 days, the inability to construct 16S rRNA sequencing libraries confirmed successful establishment of the microbiota dysbiosis model in the ABX+ EAMG group. Following initial and booster immunizations, body weight and behavioral scores were recorded every other day. Serum AChR-Ab expression levels were measured by an ELISA kit. Intestinal tissues were stained with HE to assess tissue structure, and immunofluorescence was performed to detect colon Claudin-1 and Muc2. Additionally, flow cytometry was used to quantify Th17 and Treg cells from the spleens.

### 2.5 Specimen collection

The morning feces of the rats were collected using a sterile fecal collection tube. The samples were promptly placed in liquid nitrogen and then transferred to a -80°C refrigerator for storage.

Before collecting blood samples, cardiac blood was drawn from rats anesthetized with isoflurane using sterile syringes and collected in sterile collection vessels for subsequent serological testing.

### 2.6 Establish the animal model of microbiota dysbiosis

Vancomycin (5 mg/ml) (MB1260, Meilunbio, Dalian, China), Neomycin (10 mg/ml) (MB1716, Meilunbio, Dalian, China), Metronidazole (10 mg/ml) (MB2200, Meilunbio, Dalian, China) and Ampicillin (10 mg/ml) (MB1260, Meilunbio, Dalian, China) were dissolved in distilled water to prepare an antibiotic mixture. The rats received the antibiotic mixture by gavage at a dose of 1 ml per 100 g body weight, administered every 12 hours for 14 consecutive days. Feces were collected on the 14th day and PCR analysis was used to construct the database. Failure to successfully construct the microbial database indicated a significant reduction in gut microbiota, confirming the successful establishment of the animal model of microbiota dysbiosis.

### 2.7 Fecal microbiota transplantation

Before transplantation into the recipient rats, fecal samples from the same group were mixed together into one sample in the anaerobic tent. This ensured a high diversity of the donor microbiota, which helped detect changes following fecal microbiota transplantation. The mixed fecal samples were then individually placed into 50 ml centrifuge tubes. After weighing, an appropriate amount of aseptic PBS solution was added to achieve a fecal concentration of 200 mg/ml. The entire preparation process was carried out on ice to maintain sample integrity. The mixture was allowed to stand for 2 hours to fully dissolve the feces, followed by shaking and mixing. The samples were centrifuged at 500 g for 10 minutes at 4°C. Discard the sediment and retain the suspension. After centrifugation at 2000 g for 5 minutes, sterile PBS, and 20% sterile glycerol were added to the suspension and mixed thoroughly (Bokoliya et al., 2021). The bacterial concentration was measured by turbidimetry and adjusted to 10^9^CFU/ml, then frozen at -80°C for later use. A dose of 1.0 ml per 100 g body weight was administered orally once daily for 7 consecutive days.

### 2.8 16S rRNA gene sequence analysis

Each sample weighed 0.5 g and nucleic acid was extracted using the OMEGA Soil DNA Kit (M5635-02, Omega Bio-Tek, Norcross, GA, USA). The size of DNA molecules was determined by 0.8% agarose gel electrophoresis, followed by quantitative analysis of DNA concentration. Forward primer 338F (5’-ACTCCTACGGGAGGCAGCA-3’) and reverse primer 806R (5’-GGACTACHVGGGTWTCTAAT-3’) were used to amplify the V3-V4 region of the bacterial 16S rRNA gene. PCR products were quantified using a specific Quantitative Assay Kit. After library construction, quality control and quantitative analysis were conducted, followed by bioinformatic analysis of the microbiome using QIIME2 version 2022.11. The taxonomic annotation of ASVs (Amplicon Sequence Variant, ASV) was performed using the Greengenes2 database, and R software was used to generate column charts. QIIME 2 and R software were employed to analyze alpha diversity indices (Chao1 and Shannon indices), beta diversitymetrics, taxonomic composition, and differential abundance between groups, with corresponding figures generated. Additionally, PICRUSt2, combined with the MetaCyc and KEGG databases, was used to predict the metabolic functions of microbial communities.

### 2.9 Hematoxylin and eosin staining

Colon tissue from rats anesthetized with isoflurane was fixed in 4% paraformaldehyde for 24 hours. The tissues were dehydrated through graded ethanol, cleared with xylene, embedded in paraffin, sectioned, and stained with hematoxylin. The reverting blue solution was reversed and eosin staining was carried out. Then the samples were dehydrated again, cleared with xylene, and sealed. Microscopic (Nikon ECLIPSE E100) examination and image analysis were then performed.

### 2.10 Immunofluorescence

Paraffin sections were dewaxed and subjected to antigen retrieval, then bovine serum albumin (BSA) was used as a blocking agent. The sections were incubated overnight at 4°C with primary antibodies (Claudin 1, 1:500, O95832, Servicebio, Wuhan, China) (MUC 2, 1:300, Q80Z19, Servicebio, Wuhan, China). The slides were further incubated with a fluorescently labeled secondary antibody at room temperature in the dark for 50 minutes. Subsequently, they were stained with 4’,6-diamidino-2-phenylindole dihydrochloride (DAPI) for 10 minutes to visualize the nuclei. Finally, the slides were observed using a fluorescence microscope.

### 2.11 Flow cytometry analysis

After the rats were anesthetized with isoflurane, spleen tissue was excised, ground and filtered. The suspension was centrifuged at 300 g for 5 minutes, and the pellet was collected. Then, 1× red blood cell lysate was added and incubated for 10 minutes. Afterward, PBS was added to terminate the lysis. The mixture was centrifuged again at 300 g for 5 minutes, the supernatant discarded, and the cell concentration adjusted to 10^6^ cells/ml. Single-cell suspensions prepared from the spleen were resuspended in cell staining buffer (eBioscience, San Diego, CA, USA). The cells were stimulated with BD leukocyte activation cocktail (BD Pharmingen, San Diego, CA, USA) at 37 °C for 4 h, Lymphocytes were collected and surface-stained with FITC Mouse Anti-Rat CD3 (BD Pharmingen, San Diego, CA, USA, clone number: G4.18), PE-Cy7 Mouse Anti-Rat CD4 (BD Pharmingen, San Diego, CA, USA, clone number: OX-35), and ANTI-RT CD25 OX39 APC (Thermo, clone number: OX39). The cells were incubated with the antibodies in the dark at 4°C. Subsequently, fixation/permeabilization solution (BD Pharmingen, San Diego, CA, USA) was added to permeabilize the membrane. For intracellular staining, ANTI-MO/RT IL-17A BV650 (ThermoFisher, Carlsbad, CA, USA, Clone number: eBio17B7) and ANTI-M/R FOXP3 FJK-16S PE (ThermoFisher, Carlsbad, CA, USA, clone number: FJK-16s) were added and incubated at 4°C in the dark. After staining, centrifuge, wash, discard supernatant, and add an appropriate amount of cell staining buffer to re-suspend cells. The populations of CD3+CD4+IL-17A+Th17 cells and CD3+CD4+CD25+Foxp3+Treg cells were identified and quantified by multiparameter flow cytometer (Apogee, Hemel Hempstead, England, United Kingdom). FlowJo software version 10.8.1 was used for analysis. Flow cytometry gating strategy shown in **Supplementary Figure 1**, **Supplementary Figure 2**.

Statistical Analysis: The GraphPad Prism 8.3.0 software is used for statistical analysis. The data are reshaped as the average value ± SD based on at least three independent experiments. Student’s t-tests are used to compare data between two groups, and one-way ANOVA is applied for comparisons among three groups. The *p*-value below 0.05 is considered statistically significant.

## 3 Results

### 3.1 Comparison of EAMG and HC

Primary immunization was administered on day 0 of the experiment. Booster immunization followed on day 30. On day 66, severe limb weakness and a moribund state appeared in the EAMG group, leading to the termination of the experiment (n=6).

**Figure 1:**
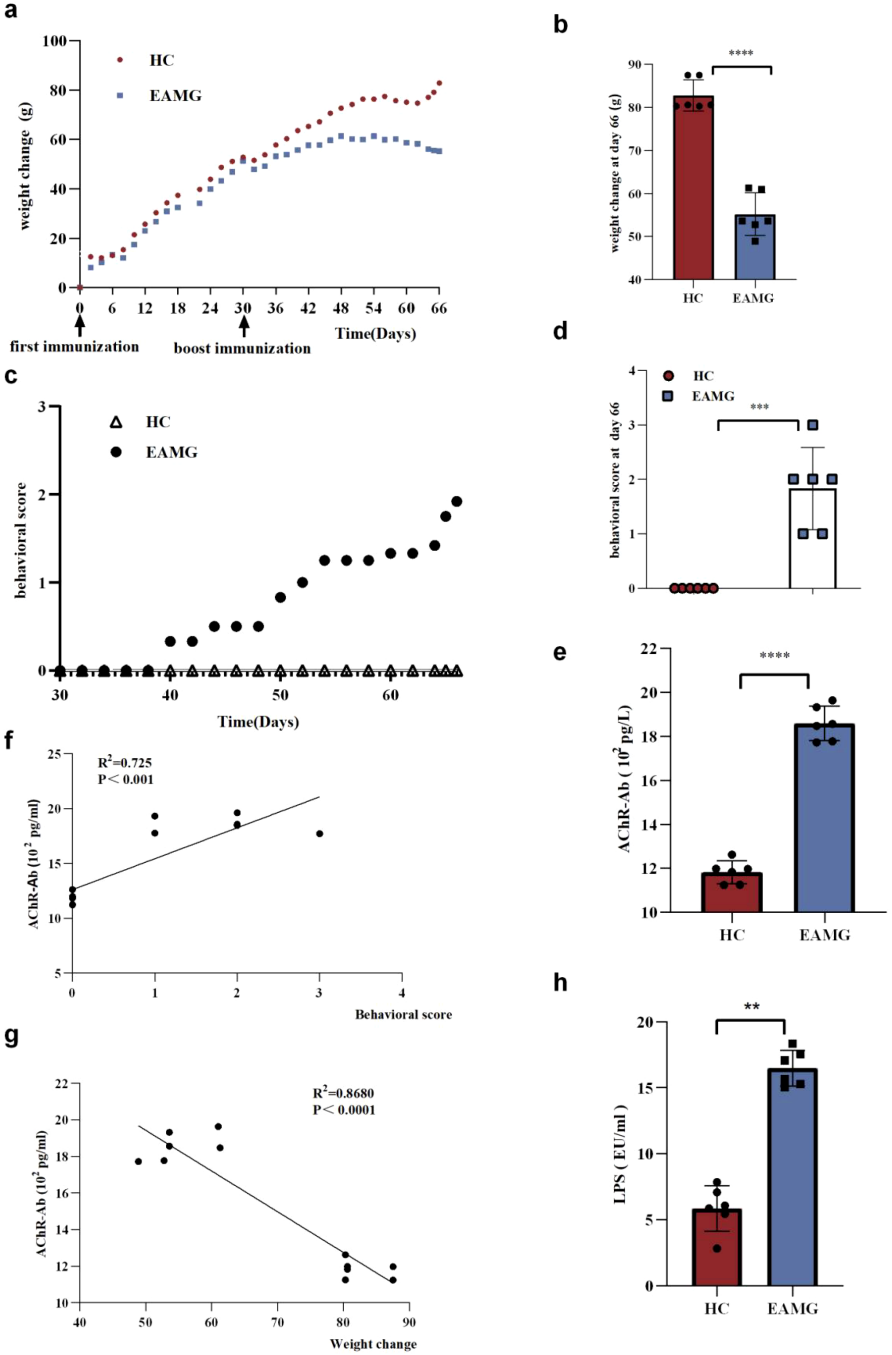
**(a)** The figure shows the mean weight changes of healthy controls (HC) and experimental autoimmune myasthenia gravis (EAMG) groups throughout the experiment. **(b)** On day 66, a significant difference in weight change was observed between the HC and EAMG groups. Individual data points represent values from each rat, with horizontal bars indicating group means. **(c)** Behavioral scores were monitored in both HC and EAMG groups throughout the experimental period. **(d)** Comparison of HC and EAMG behavioral scores on day 66. **(e)** Comparison of serum AChR-Ab expression levels between HC and EAMG at the 66 day of the experiment. **(f)** A significant positive correlation was found between behavioral scores and anti-acetylcholine receptor antibody (AChR-Ab) levels (R² = 0.725, *p* < 0.001). **(g)** The change in body weight was negatively correlated with AChR-Ab levels (R^2^ = 0.8680, *p* < 0.0001). **(h)** Serum LPS expression level. (ns=no statistical significance, *p < 0.05, **p < 0.01; ***p < 0.001, ****p < 0.0001).

Weight changes in the EAMG group exhibited a trend distinct from that of the HC group (**Figure 1a**). The HC group showed gradual weight gain, while the EAMG group exhibited initial slow growth followed by progressive weight loss after booster immunization. On day 66, the EAMG group’s body weight was significantly lower than that of the HC group (**Figure 1b**, *p* < 0.0001). Behavioral score comparisons between the groups (**Figure 1c**) revealed progressively increasing scores in the EAMG group following immunization. On day 66, behavioral scores in the EAMG group were significantly higher than in the HC group (**Figure 1d**, *p* < 0.001).

Serum ELISA tests conducted on day 66 demonstrated significantly higher AChR-Ab levels in the EAMG group compared to the HC group (**Figure 1e**, *p* < 0.0001). These results confirmed the successful establishment of the EAMG model. Furthermore, body weight and behavioral scores differed significantly between the HC group and the EAMG group. 

We also observed a positive correlation between behavioral scores and serum AChR-Ab levels (**Figure 1f**). In contrast, body weight changes showed an inverse relationship with AChR-Ab expression (**Figure 1g**). These findings indicate that both AChR-Ab expression level and body weight can serve as indicators of EAMG severity.

**Figure 2:**
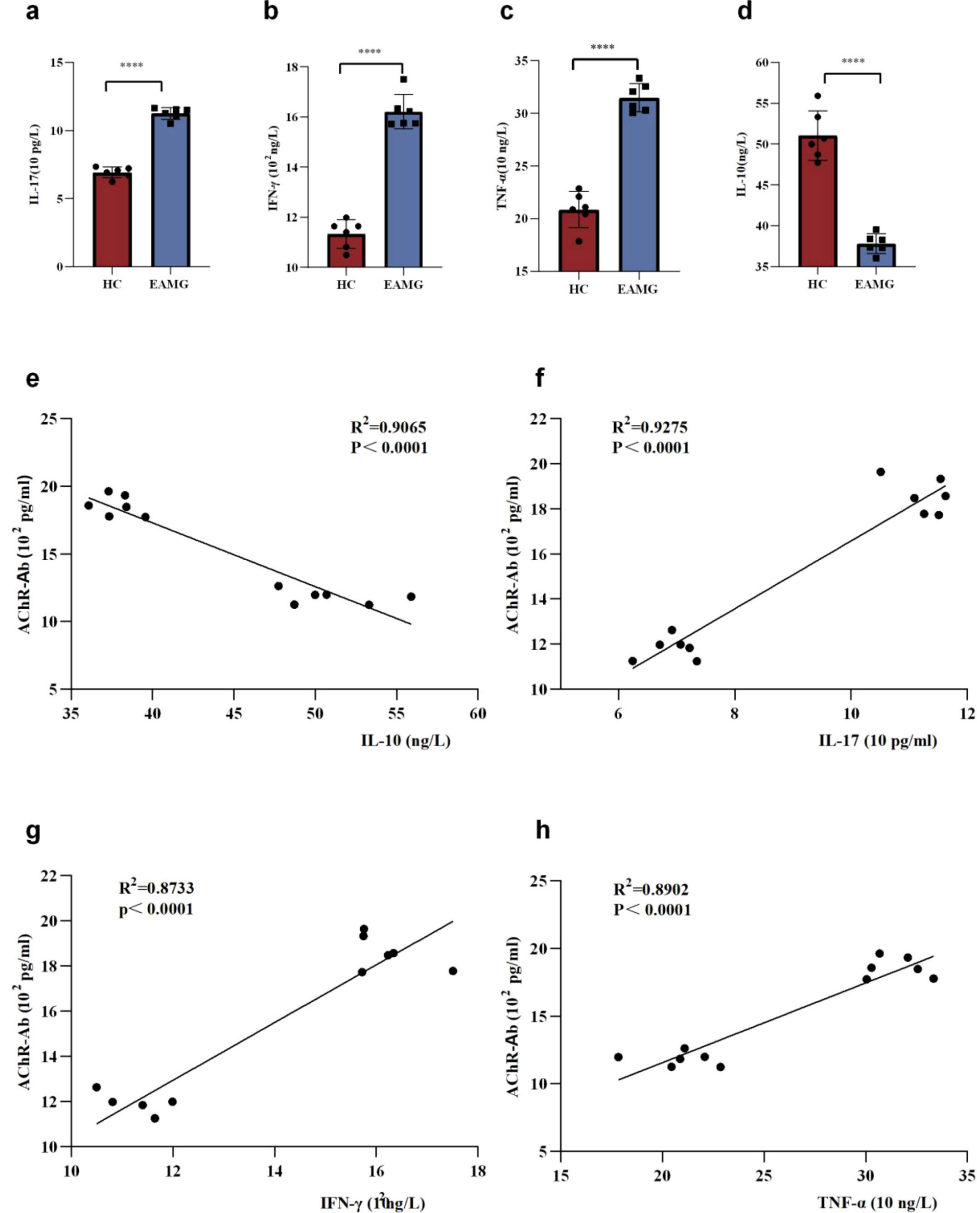
**(a)** Serum IL-17 levels showed marked elevation in EAMG compared with HC. **(b)** A significant increase in IFN-γ levels was observed in the EAMG group versus the HC group. **(c)** The TNF-α level difference between EAMG and HC reached statistical significance (*p* < 0.0001). **(d)** The serum IL-10 expression level of EAMG was significantly lower than that of the HC group. **(e)** A strong negative correlation was observed between serum IL-10 levels and AChR-Ab titers (R^2^ = 0.9065, *p* < 0.0001). **(f)** Serum IL-17 expression level was positively correlated with AChR-Ab (R^2^ = 0.9275, *p* < 0.0001). **(g)** Serum IFN-γ expression level was positively correlated with AChR-Ab (R^2^ = 0.8733, *p* < 0.0001). **(h)** The TNF-α expression level was positively correlated with AChR-Ab (R^2^ = 0.8902, *p* < 0.0001).(ns=no statistical significance, *p < 0.05, ** *p* < 0.01; *** *p* < 0.001, **** *p <*0.0001).

Inflammation plays a critical role in the pathogenesis of EAMG. At the end of the experiment, serum inflammatory cytokines IL-10, IL-17, IFN-γ and TNF-α expression levels in EAMG and HC groups were measured by ELISA. We found that serum IL-17, IFN-γ, and TNF-α levels in the EAMG group were significantly higher than those in the HC group (**Figures 2a–c**) (*p* < 0.0001), while serum IL-10 levels were significantly lower than those in the HC group (**Figure 2d**) (*p* < 0.0001). Correlation analysis between inflammatory cytokines and serum AChR-Ab revealed that IL-10 levels in EAMG were inversely correlated with AChR-Ab levels, whereas IL-17, IFN-γ, and TNF-α levels showed a positive correlation (**Figures 2e–h**). IL-17, IFN-γ, and TNF-α exhibited pro-inflammatory effects and exacerbated tissue immune damage. In contrast, IL-10 possessed anti-inflammatory properties and mitigated immune tissue damage. Our results suggest that inflammation is involved in the pathogenesis of EAMG. Additionally, we also found that the serum levels of LPS in EAMG serum were significantly increased (*p* < 0.001) (**Figure 1h**). This is a direct indication of the leakage of bacterial components from the intestinal tract into the bloodstream. An increase in serum LPS levels reflects increased intestinal permeability.

**Figure 3:**
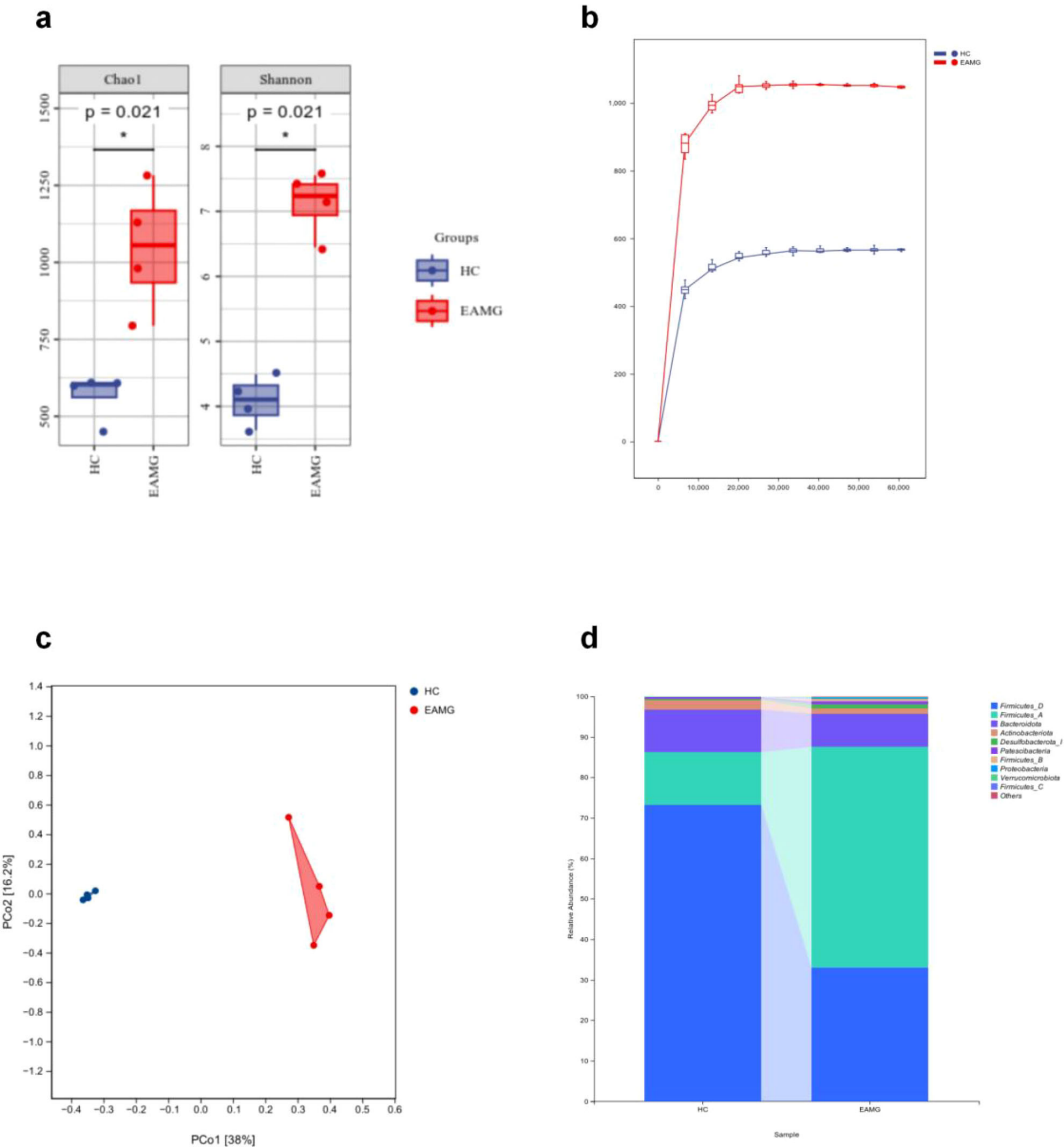
**(a)** Statistically significant differences were observed between the Chao1 and Shannon indices (n=4). **(b)** Sparse curve analysis revealed distinct α-diversity patterns in gut microbiota between groups (n=4). **(c)** PCoA demonstrated significant β-diversity separation between HC and EAMG groups (n=4). **(d)** Significant phylum-level compositional disparities were identified in gut microbiota between HC and EAMG groups. (*p < 0.05).

Changes in the diversity and composition of gut microbiota were observed in EAMG. Fecal samples from rats in the two groups were sequenced using 16S rRNA gene sequencing (n=4). Significant differences in the Chao1 and Shannon indices existed between the groups, with higher values indicating increased alpha diversity in the EAMG group (**Figure 3a**, *p* < 0.05). At the same sequencing depth, the ASV/OTU (Operational Taxonomic Unit, OTU) count and alpha diversity were significantly higher in the EAMG group than in the HC group (**Figure 3b**, *p* < 0.05). Principal coordinates analysis (PCoA) revealed distinct clustering along the coordinate axes (**Figure 3c**). This result further confirmed differences in beta diversity between the HC and EAMG groups.

**Figure 4:**
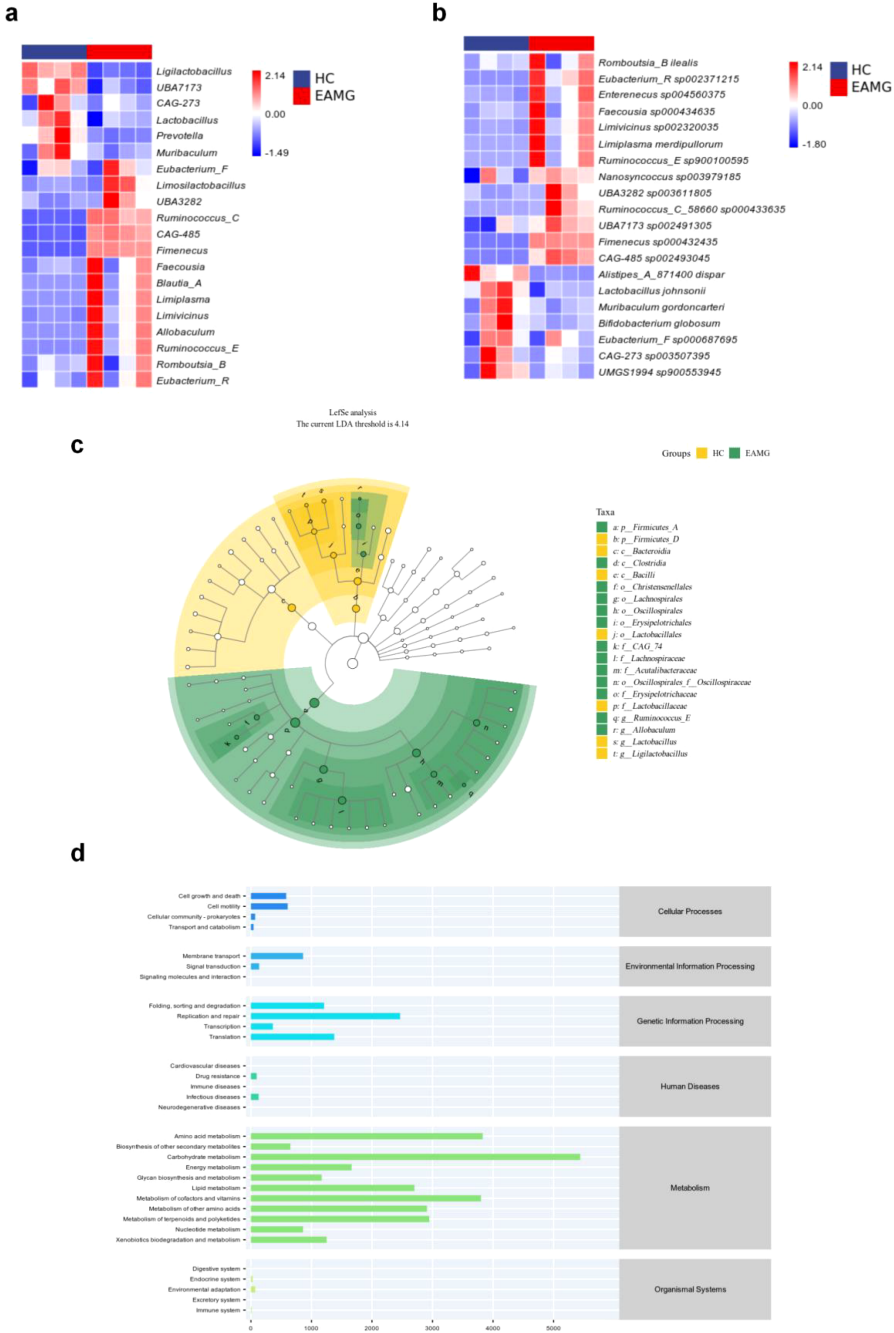
**(a)** The heat map of gut microbiota composition at the genus level. **(b)** The heatmap of gut microbiota composition at the species level. **(c)** LEfSe analysis of gut microbiota. The main role of gut microbiota in EAMG is *Firmicutes_*A and *Clostridia*, while the main role of gut microbiota in HC is *Firmicutes*_D and *Bacteroidia* (n=4). **(d)** Prediction of metabolic pathways in gut microbiota. The KEGG pathway was obtained from microbiota analysis data using PICRUSt.

Analysis of taxonomic composition revealed that, at the phylum level, *Firmicutes* and *Bacteroidota* dominated the gut microbiota in the HC group (**Figure 3d**). Within *Firmicutes*, two major subgroups-referred to as *Firmicutes* D (73.24%) and *Firmicutes* A (13.01%)-were the most abundant, followed by *Bacteroidota* (10.52%). By comparison, the EAMG group exhibited a higher proportion of *Firmicutes* subgroup A (54.48%) and lower proportions of *Firmicutes* subgroup D (33.06%) and *Bacteroidota* (8.17%). Additionally, the relative abundance of *Actinobacteriota* was lower in EAMG (1.30%) compared to HC (2.39%). These findings indicate an increased *Firmicutes* A and decreased *Firmicutes* D, *Bacteroidota*, and *Actinobacteriota* in EAMG, reflecting distinct microbial composition between the groups. At the genus level, heatmap comparisons revealed that the genus *Ruminococcus* C was significantly increased in EAMG rats, whereas the genus *Ligilactobacillus* was decreased (**Figure 4a**). At the species level, *Lactobacillus johnsonii* and *Bifidobacterium globosum* were markedly decreased in EAMG rats but increased in HC rats (**Figure 4b**). Linear discriminant analysis Effect Size (LEfSe) analysis (**Figure 4c**) showed that *Firmicutes* A and the class *Clostridia* dominated the gut microbiota of EAMG rats, while *Firmicutes* D and the class *Bacteroidia* dominated in HC. These results further confirmed distinct gut microbiota profiles between EAMG and healthy rats. KEGG metabolic pathway analysis (**Figure 4d**) revealed that EAMG was associated with changes in the abundances of microbial genes involved in pathways related to metabolism, genetic information processing, cellular processes, environmental information processing, human diseases, and organismal systems. These findings suggest that the gut microbiota are closely associated with various aspects of host physiological functions. In summary, the composition and diversity of the gut microbiota in EAMG rats were significantly altered compared to healthy Lewis rats. Such changes indicate dysbiosis of the gut microbiota and simply a potential impact on host physiological systems.

**Figure 5:**
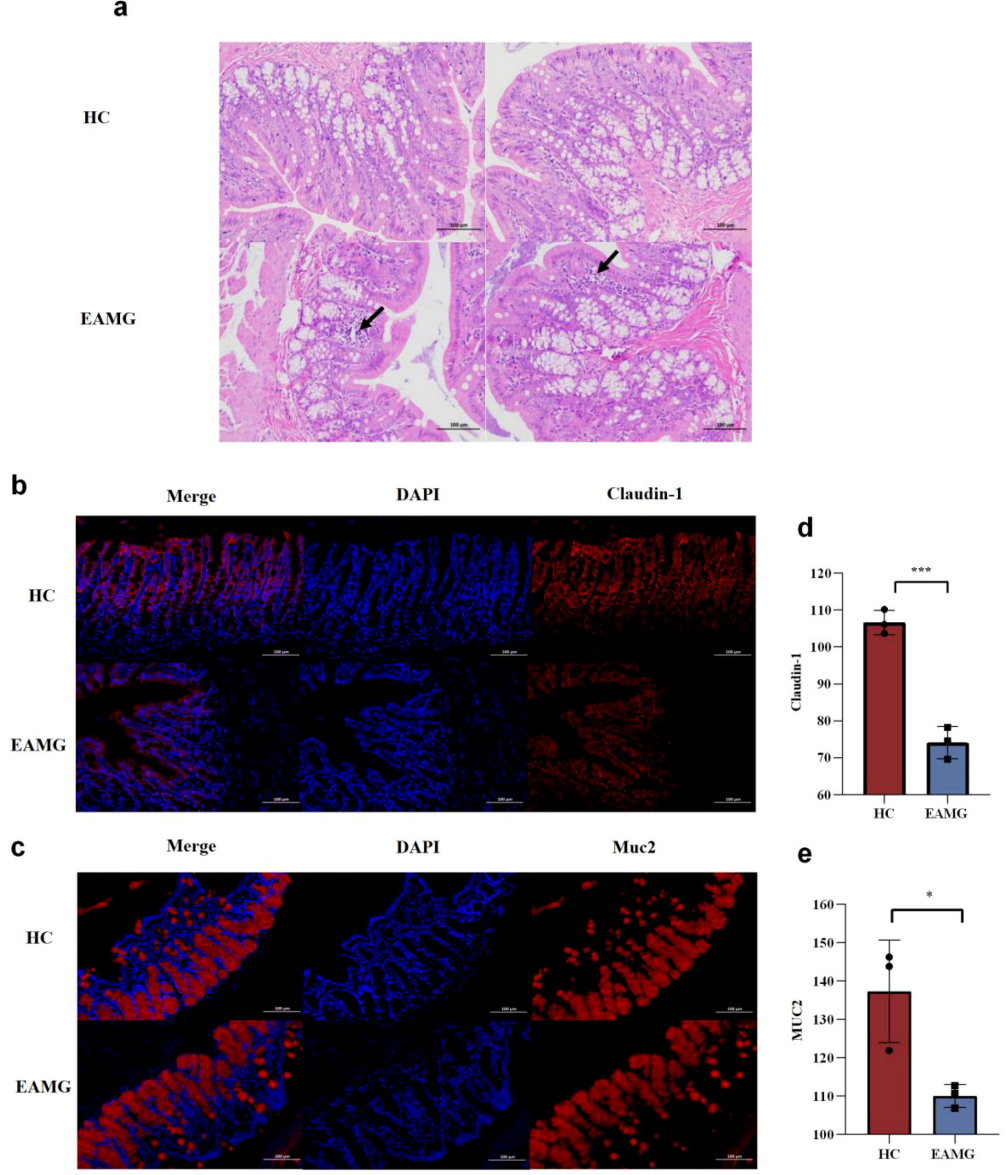
**(a)** The mucosal layer of colon tissue in the HC group was characterized by multiple folds, and the mucosal epithelium was mainly composed of single columnar epithelial cells and goblet cells. Lamina propria has abundant intestinal glands, which are round or long tubes, and goblet cells are abundant. In EAMG group, a small number of lymphocytes could be observed as focal aggregation (black arrow) and scattered distribution. **(b)** Representative image of colon with Claudin-1 immunofluorescence staining in HC and EAMG groups. (blue: nucleus; red: claudin-1, scale bar: 100 μm) **(c)** Representative image of colon Muc2 immunofluorescence staining in HC group and EAMG group.(blue: nucleus; red: Muc2 scale bar: 100 μm) **(d)** The expression of intestinal mucosal tight junction protein Claudin-1 in the HC group was significantly higher than that in the EAMG group. **(e)** The expression of intestinal Muc2 in the HC group was significantly higher than that in the EAMG group. Data were expressed as mean ± SD (n=3). (ns, no statistical significance; *p < 0.05, **p < < 0.01; ***p < 0.001, ****p < 0.0001).

The intestinal barrier of EAMG is impaired. After anesthesia, colons were collected from rats in both groups for HE staining (**Figure 5a**). The colonic mucosa of rats in the HC group showed numerous folds, abundant intestinal glands, and goblet cells in the lamina propria. In contrast, focal infiltration of colonic lymphocytes was observed in the EAMG group, indicating inflammation in the colon of these rats. To further assess the intestinal barrier, we compared the immunofluorescence staining of colonic tight junction protein Claudin-1 and mucin 2 (Muc2) between the two groups (**Figures 5b, c**). Relative fluorescence intensity was quantified using “Image J” software (n=3). The expression of Claudin-1 in colonic tissues from the EAMG group was significantly lower than that in the HC group (**Figure 5d**) (*p* < 0.001). Similarly, the expression of Muc2 in the EAMG group was significantly lower than that in the HC group (**Figure 5e**) (*p* < 0.05). The colonic tight junction protein Claudin-1 is involved in forming tight junctions that restrict macromolecular transport across intercellular spaces, thereby protecting the intestine from harmful substances (Lau et al., 2015). Muc2 is a crucial component of the intestinal mucosal barrier as it forms the mucus layer that protects the epithelium and maintains intestinal homeostasis (Rhodes, 1997). In the colon of EAMG rats, immunofluorescence analysis revealed marked inflammatory cell infiltration and reduced expression of Claudin-1 and Muc2, clearly demonstrating intestinal barrier damage.

### 3.2 The effect of fecal microbiota transplantation on Lewis rats

**Figure 6:**
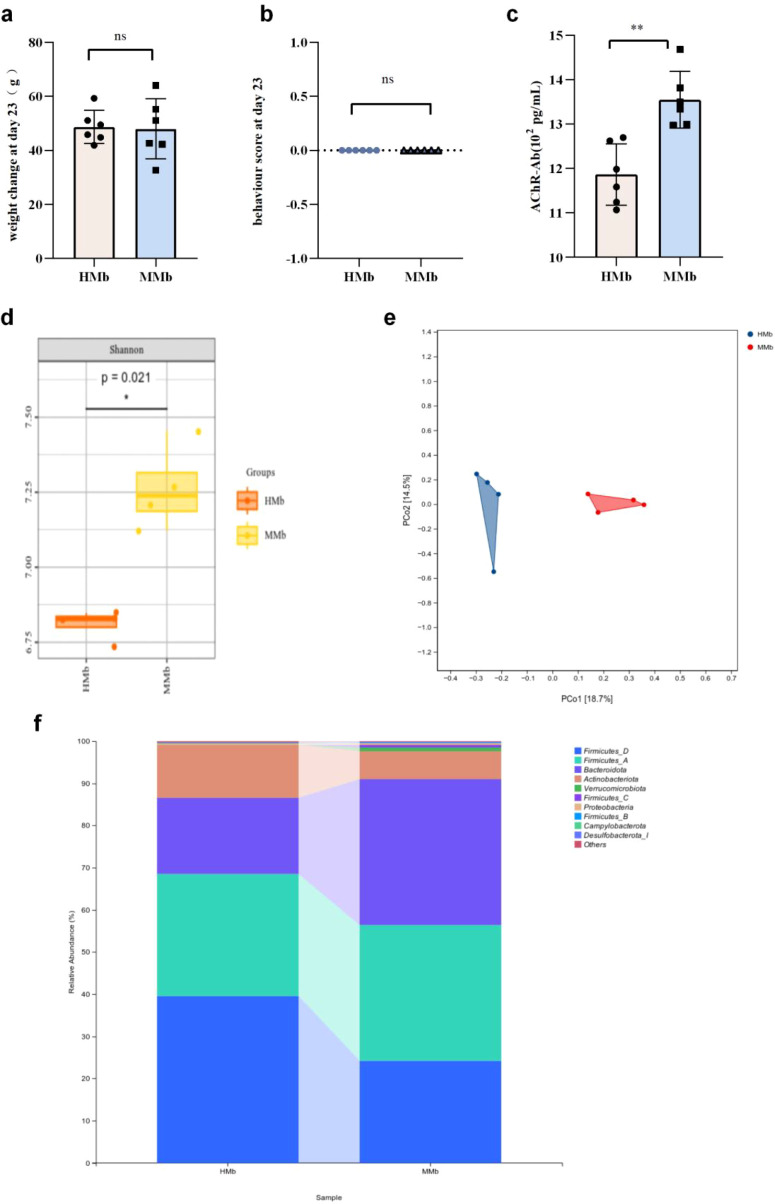
**(a)** There was no significant difference in body weight between the two groups at day 23 of the experiment (after bacterial transplantation). **(b)** There was no significant difference in behavioral scores between the HMb and MMb groups at day 23 of the experiment. **(c)** The serum AChR-Ab expression level in MMb group was significantly higher than that in HMb group. Data were expressed as mean ± SD (n=6). **(d)** There was a statistical difference in the Shannon index between the HMb group and the MMb group.**(e)** PCoA analysis revealed two distinct matrices in the HMb and MMb groups, indicating the presence of β diversity in both groups (n=4). **(f)** The composition of intestinal flora in the HMb and MMb groups was different at the phylum level. (n=4). (ns=no statistical significance, *p < 0.05, **, p < 0.01; *** *p <* 0.001, **** *p <* 0.0001).

We established microbial dysbiosis animal models by continuously gavaging rats with an antibiotic mixture for 14 days. Fecal microbiota suspensions from HC and EAMG donors were transplanted daily into Lewis rats for 7 consecutive days. Rats receiving microbiota from the HC group were named the HMb group, and those receiving EAMG microbiota were named the MMb group. Each group included six rats (n=6). After microbiota transplantation, no significant differences were observed in the weight changes or behavioral scores, which were zero in both HMb and MMb groups. There was no significant difference observed between MMb and HMb, indicating no observable symptoms (**Figures 6a, b**). Despite the absence of differences in body weight and behavior, we measured serum AChR-Ab expression levels to assess immunological changes. The serum AChR-Ab level in the MMb group was significantly higher than in the HMb group (**Figure 6c**, *p* < 0.01). We found a significant association between AChR-Ab levels and EAMG severity. Despite no differences in body weight or behavioral scores between the HMb and MMb groups, the significantly elevated AChR-Ab levels in the MMb suggest that intestinal microbiota may contribute to the pathological progression of EAMG. However, the underlying mechanisms remain to be elucidated.

There are differences in the intestinal microbial communities between HMb and MMb. After microbiota transplantation, we sequenced the gut microbiota of the HMb and MMb groups using 16S rRNA gene sequencing. The results revealed significant differences in species diversity (Shannon index) between the HMb and MMb groups (**Figure 6d**, *p* < 0.05). PCoA showed distinct clustering patterns along the principal coordinate axes, indicating significant differences in gut microbiota composition between the MMb and HMb groups. This reflects differences in beta diversity (**Figure 6e**). Moreover, the results indicated that the gut microbiota composition changed after transplantation, suggesting a successful microbiota transplantation.

**Figure 7:**
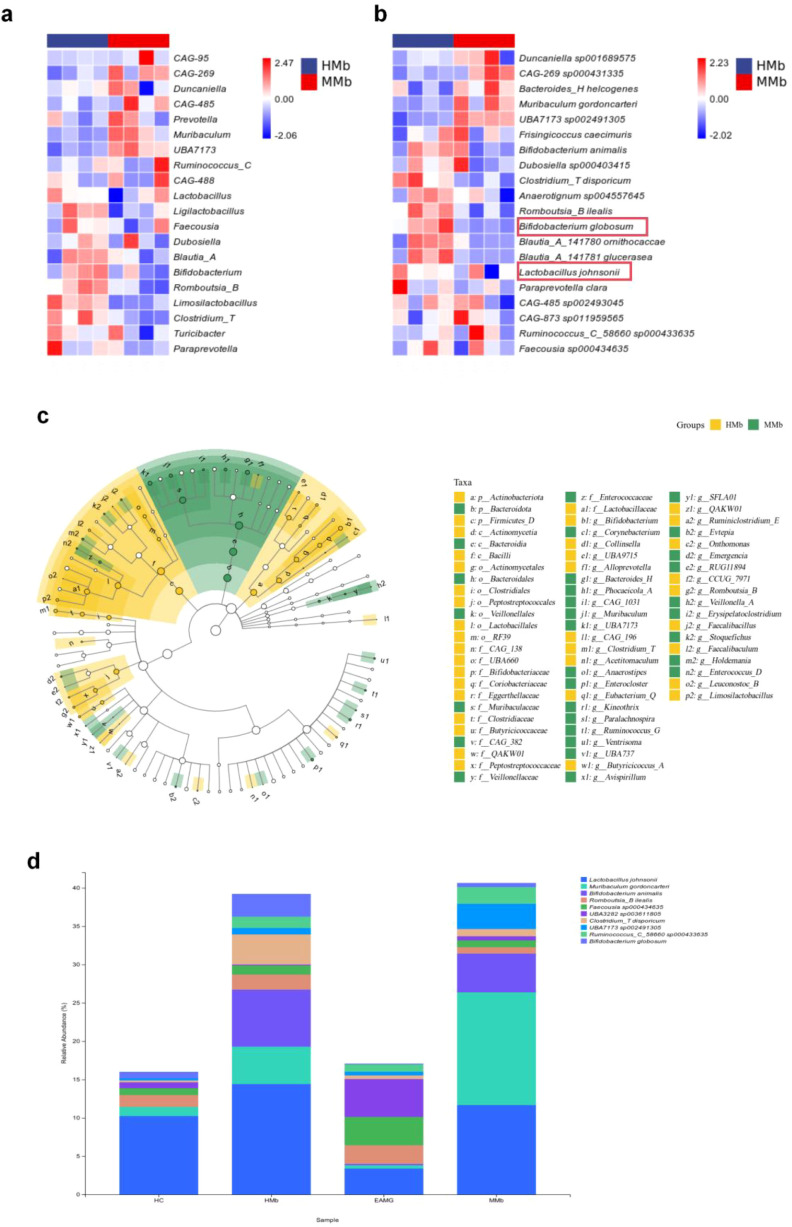
**(a)** Species composition heat maps of gut microbiota in HMb and MMb groups at the genus level (n=4). **(b)** Species composition heat maps of gut microbiota in HMb and MMb groups at the species level (n=4). **(c)** LEfSe diagram analysis of gut microbiota in HMB and MMb group. *Actinobacteriota* and *Firmicutes*_D play the main roles in the HMb group, while *Bacteroidota* plays the main role in the MMb group (n=4). **(d)** Taxonomic composition analysis was conducted on the intestinal microbiota of the four groups of rats in the recipient and donor sectors. *Muribaculum gordoncarteri* and *Lactobacillus johnsonii* were representative strains for microbiota transplantation at the species level (n=4).

Taxonomic composition analysis (**Figure 6f**) revealed differences in gut microbiota between the two groups at the phylum level. The relative abundance of *Firmicutes* D in the HMb group was significantly higher than that in the MMb group (39.61% compared to 24.18%). Conversely, *Firmicutes* A was more abundant in the MMb group than in the HMb group (28.87% in HMb and 32.20% in MMb). This difference in *Firmicutes* A abundance between the HMb and MMb groups was consistent with that observed between the HC and EAMG groups. Heat maps of taxonomic composition analysis for HMb and MMb groups showed that *Ligilactobacillus* and *Bifidobacterium* were predominant at the genus level in HMb. The relative abundance of *Limosilactobacillus* in the HMb group was significantly higher (**Figure 7a**). At the species level (**Figure 7b**), the relative abundance of *Lactobacillus johnsonii* and *Bifidobacterium globosum* decreased in the MMb group. LefSe analysis results (**Figure 7c**) showed that *Actinobacteriota* and *Firmicutes* D were dominant in the HMb group, while *Bacteroidota* was dominant in the MMb group. We conducted a taxonomic composition analysis on the intestinal microbiota from recipients and donors in four groups (**Figure 7d**). The results showed that *Muribaculum gordoncarteri* and *Lactobacillus johnsonii* were representative identified in microbiota transplantation at the species level.

**Figure 8:**
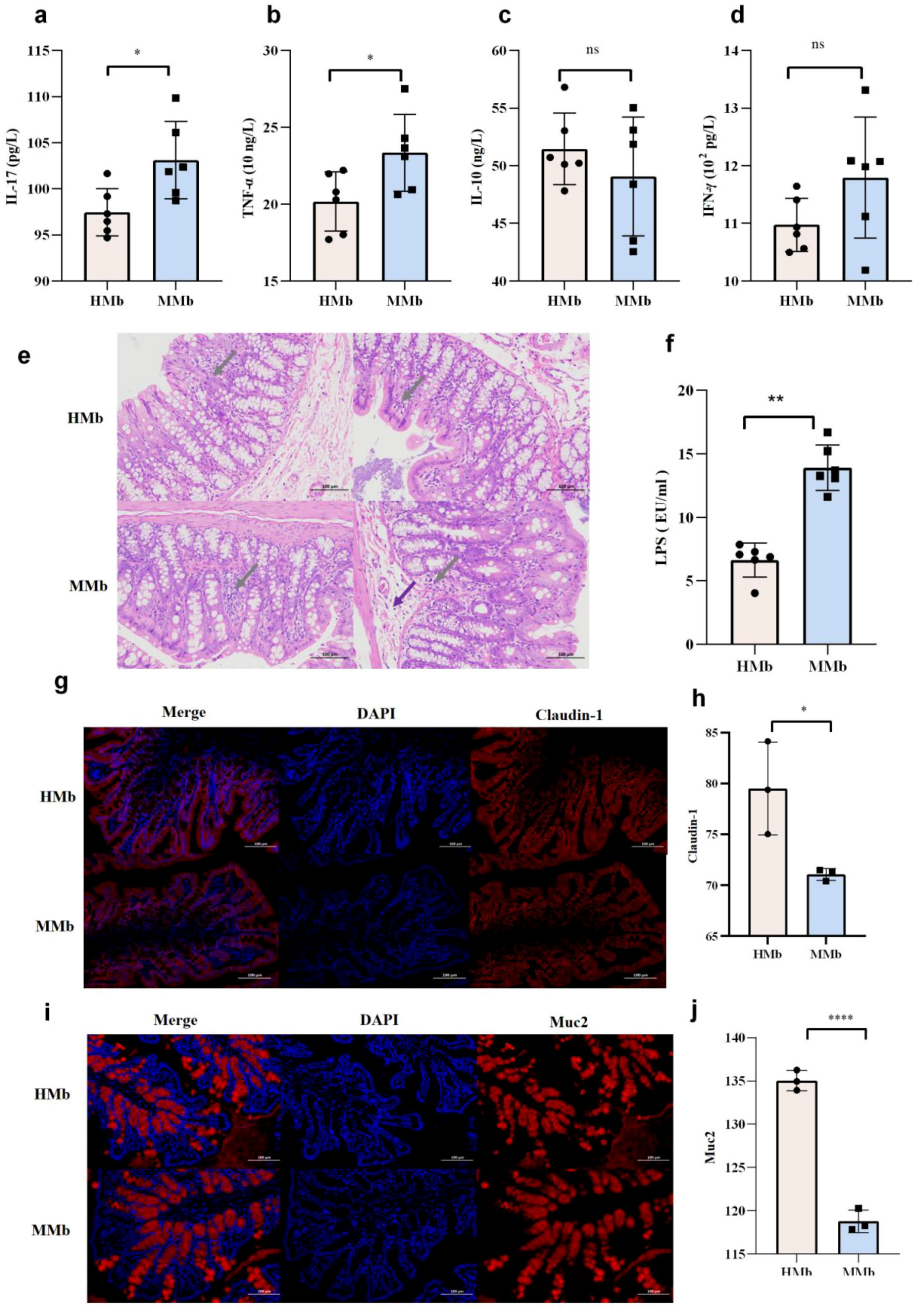
**(a)** The expression level of IL-17 in HMb group was significantly lower than that in MMb group. **(b)** The expression level of TNF-α in MMb group was significantly higher than that in HMb group. **(c)** There was no statistical analysis of IL-10 expression in HMb and MMb groups. **(d)** There was no significant difference in IFN-γ expression between HMb and MMb groups. **(e)** HE staining of colon in HMb and MMb groups. HMb group: goblet cells abundant, lymphocytes scattered distribution (gray arrow). MMb group: submucosal focal edema (purple arrow), lymphocytes scattered distribution (gray arrow). There were more inflammatory cells in the colon of MMb group than HMb group. **(f)** The expression level of serum LPS in the MMb group was significantly higher than that in the HMb group. **(g)** Representative image of colon with Claudin-1 immunofluorescence staining in HMb and MMb groups. (blue: nucleus; red: claudin-1, scale bar: 100 μm). **(h)** The expression of intestinal mucosal tight junction protein Claudin-1 in the HMb group was significantly higher than that in the MMb group. **(i)** Representative image of colon Muc2 immunofluorescence staining in HMb group and MMb group (blue: nucleus; red: Muc2; scale bar: 100 μm). **(j)** The expression of Muc2 in the HMb group was significantly higher than that in the MMb group. Data were expressed as mean ± SD (n=3). (ns, no statistical significance; *p < 0.05, **p < 0.01; ***p < 0.001, ****p < 0.0001).

The gut microbiota associated with EAMG promoted systemic inflammatory responses. Serum inflammatory cytokines in the HMb and MMb groups were measured by ELISA. The results showed that serum IL-17 and TNF-α levels in the MMb group were significantly higher than the levels in the HMb group (**Figures 8a, b**) (*p* < 0.05). We observed no significant differences in the expression levels of IL-10 and IFN-γ between the MMb and HMb groups (**Figures 8c, d**). Collectively, these findings indicate that EAMG-induced gut microbiota dysbiosis promotes systemic inflammatory responses, as evidenced by significantly elevated levels of the pro-inflammatory cytokines IL-17 and TNF-α.

Gut microbiota of EAMG impair intestinal barrier function. After bacterial transplantation, colon tissues were collected for HE staining. In the MMb group, intestinal mucosal lymphocytes were scattered and focal edema was observed in the submucosa (**Figure 8e**). In the HMb group, the intestinal glands in the lamina propria were abundant and showed round or tubular shapes. Additionally, abundant goblet cells were present, and lymphocytes were sparsely distributed. The histological findings indicated that inflammatory cell infiltration was more pronounced in the colon of the MMb group than in that of the HMb group. Correspondingly, the expression level of serum LPS in the MMb group was significantly higher than that in the HMb group (*p* < 0.01) (**Figure 8f**). This result emphasizes the crucial role of intestinal microbiota composition in maintaining intestinal barrier integrity. The tight junction protein Claudin-1 and Muc2 in colon tissue were detected by immunofluorescence staining, with representative images shown in **Figures 8g, i**. Relative fluorescence intensity was quantified using “Image J” software (n=3). The results show that the mean expression levels of Claudin-1 (*p* < 0.05) and Muc2 (*p* < 0.0001) are significantly higher in the HMb group than in the MMb group (**Figures 8h, j**). These findings indicate that alterations in the intestinal microbial community of EAMG rats are associated with damage to the colon barrier. We used both immunofluorescence and HE staining to detect and clearly visualize the intestinal tissue structure. We analyzed that the altered intestinal microbiota in EAMG rats was linked to damage in both the intestinal tissue structure and the mucosal barrier. These pathological changes contributed to elevated serum AChR-Ab titers and exacerbated disease progression.

### 3.3 The impact of microbiota dysbiosis on EAMG

The experimental animals were randomly allocated to three groups (n=6): healthy control (HC), experimental autoimmune myasthenia gravis (EAMG), and antibiotic-treated EAMG (ABX+EAMG). The ABX+EAMG group was treated with an antibiotic mixture for 14 days to induce microbiota dysbiosis. Subsequently, the primary immunization was administered on Day 14, followed by a booster immunization on Day 44.

**Figure 9:**
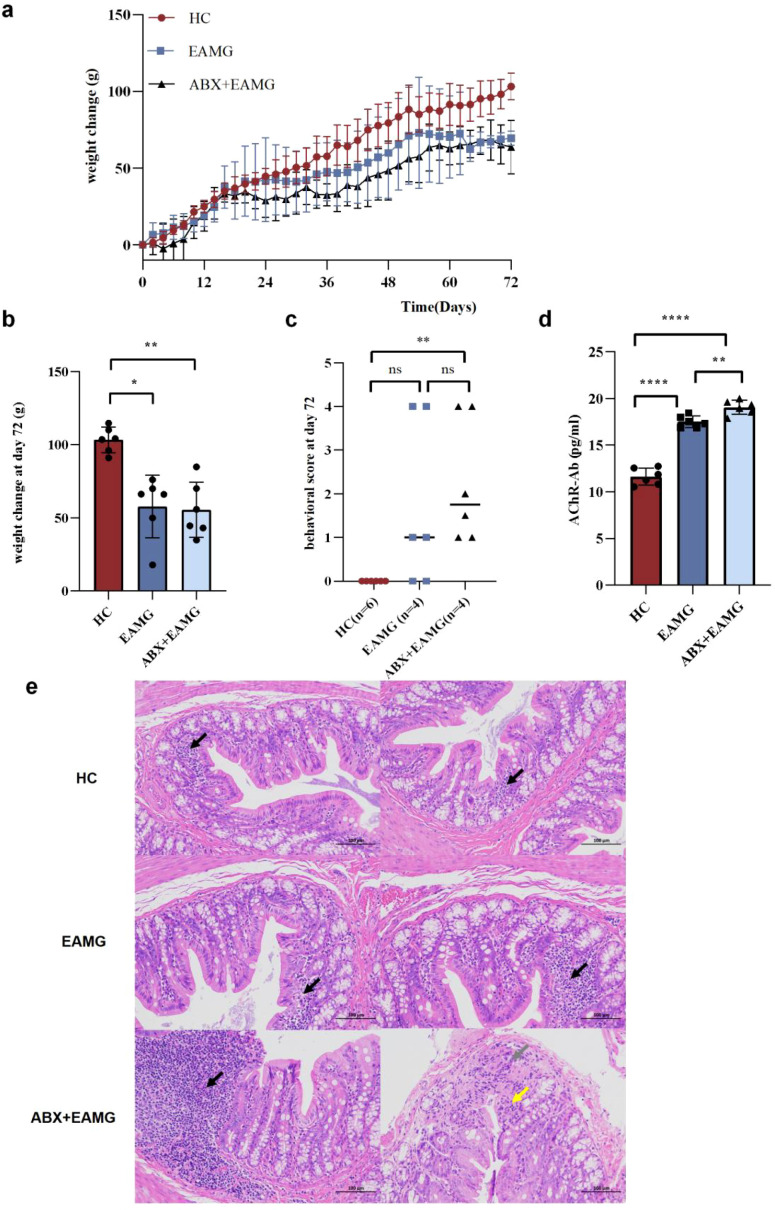
**(a)** The figure showed that the weight gain was the fastest in the HC group and the slowest in the ABX+EAMG group (n=6). **(b)** At day 72, the weight changes of the three groups were compared. The results showed that body weight in the HC group increased the fastest, significantly higher than that in the EAMG group and ABX+EAMG group. However, there was no statistical difference between the EAMG group and the ABX+EAMG group. **(c)** The behavioral scores of the three groups at day 72 were compared, and the results showed that the behavioral scores of the HC group were significantly higher than those of the ABX+EAMG group. **(d)** Compared the serum AChR-Ab of the three groups, the serum AChR-Ab expression level in the ABX+EAMG group was significantly higher than that in the HC group and EAMG group. Data was expressed as mean ± SD (n=6). **(e)** HE staining images of colon in HC group, EAMG group, ABX+EAMG group. HC group: There are a large number of intestinal glands in the lamina propria with loose arrangement, a large number of goblet cells, and a small number of lymphocytes with focal aggregation (black arrow). EAMG group: There were a large number of intestinal glands in the lamina propria, a large number of goblet cells, and more lymphocytes and macrophages clustered focally (black arrow); The mucosal muscle layer separates the lamina propria from the submucosa. Sporadic infiltration of lymphocytes in submucosa (yellow arrow). ABX+EAMG group: Focal aggregation of numerous lymphocytes and macrophages (black arrow). Focal erosion can be seen in lamina propria (gray arrow), mucosal epithelium and intestinal gland necrosis disappear, a small amount of necrotic cell debris can be seen, a small amount of fibrous connective tissue hyperplasia, with a small amount of lymphocyte infiltration (yellow arrow). As shown in the figure, the ABX+EAMG group had the highest number of inflammatory cells in intestinal tissue. (ns, no statistical significance; *p < 0.05, **p <0.01, ***p < 0.001, ****p <0.0001).

Gut microbiota dysbiosis aggravates myasthenia symptoms in EAMG. Body weight trends (**Figure 9a**) demonstrated that the HC group gained weight fastest, while the ABX+EAMG group showed the slowest weight gain progression. The HC group showed significantly greater weight gain at the endpoint (Day 72) than both the EAMG group (*p* < 0.05) and the ABX+EAMG group (*p* < 0.01) (**Figure 9b**). The behavioral scores of all groups at the endpoint (**Figure 9c**) revealed that the ABX+EAMG group had significantly worse scores compared with the HC group (*p* < 0.01). Serum AChR-Ab concentration was measured by ELISA on Day 72 after the initial immunization, revealing that the concentration in the ABX+EAMG group was significantly higher than that in the EAMG group (*p* < 0.01) and the HC group (*p* < 0.0001). These results demonstrate that, compared to both HC and EAMG groups, gut microbiota dysbiosis induced in AChRα97–116 peptide-immunized Lewis rats causes three key effects: slower body weight gain, earlier onset of limb weakness, and higher serum AChR-Ab levels.

**Figure 10:**
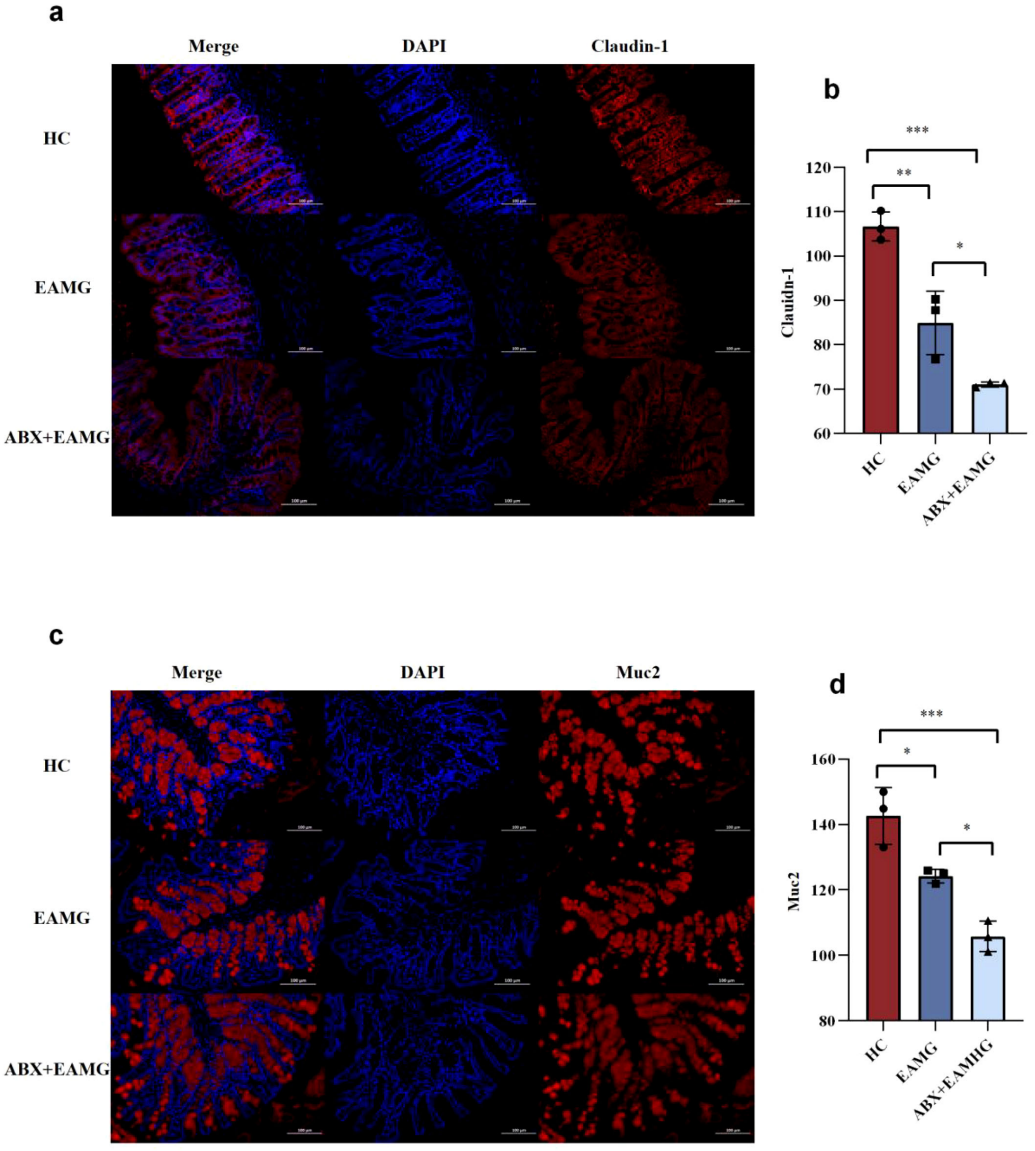
**(a)** Representative immunofluorescence staining of the colon tight junction protein Claudin-1 in HC, EAMG, and ABX+EAMG group. (blue: nucleus; red: claudin-1, scale bar: 100 um). **(b)** The mean Claudin-1 density in the HC group was higher than that in the EAMG group and ABX+EAMG group, and the Claudin-1 density in the ABX+EAMG group was significantly lower than that in the EAMG group, indicating that the Claudin-1 density in the colon of EAMG with flora disorder (ABX+EAMG group) was significantly reduced. Data was expressed as mean ± SD (n=3). **(c)** Representative immunofluorescence staining of the Muc2 of the colon in HC, EAMG, and ABX+EAMG group. (blue, nucleus; red, Muc2; scale bar: 100 μm). **(d)** The mean Muc2 density in the HC group was higher than that in the EAMG group and ABX+EAMG group. Furthermore, the Claudin-1 density in the ABX+EAMG group was significantly lower than that in the EAMG group, indicating that the colonic Muc2 expression of EAMG with flora disorder (ABX+EAMG) was significantly reduced. Data was expressed as mean ± SD (n=3). (ns, no statistical significance; *p < 0.05, **p < 0.01, ***p < 0.001, ****p <0.0001).

Gut microbiota dysbiosis impairs the EAMG intestinal barrier. In **Figure 9e**, HE staining of rat colon showed focal clusters of lymphocytes and macrophages in the EAMG group. Compared with the HC group, inflammatory infiltration in intestinal tissue was more significant in rats with microbiota disturbance (ABX+EAMG group), whereas only a few lymphocyte foci appeared in the HC group. Overall, inflammatory infiltration in intestinal tissue was significantly higher in the ABX+EAMG group. This indicates that gut microbiota dysbiosis can worsen inflammatory cell infiltration in colon tissue and promote intestinal damage. Immunofluorescence staining of the intestinal tight junction protein Claudin-1 is shown in **Figure 10a**. Relative fluorescence intensity was quantified using “Image J” software (n=3). It showed that Claudin-1 expression was lowest in the ABX+EAMG group and highest in the HC group (**Figure 10b**), and the difference was statistically significant. As shown in **Figures 10c, d**, immunofluorescence staining revealed a significant reduction of Muc2 expression in the ABX+EAMG group, consistent with the Claudin-1 findings. This marked decrease in both proteins indicates that gut microbiota disruption compromises intestinal barrier integrity by downregulating tight junction proteins and mucins.

**Figure 11:**
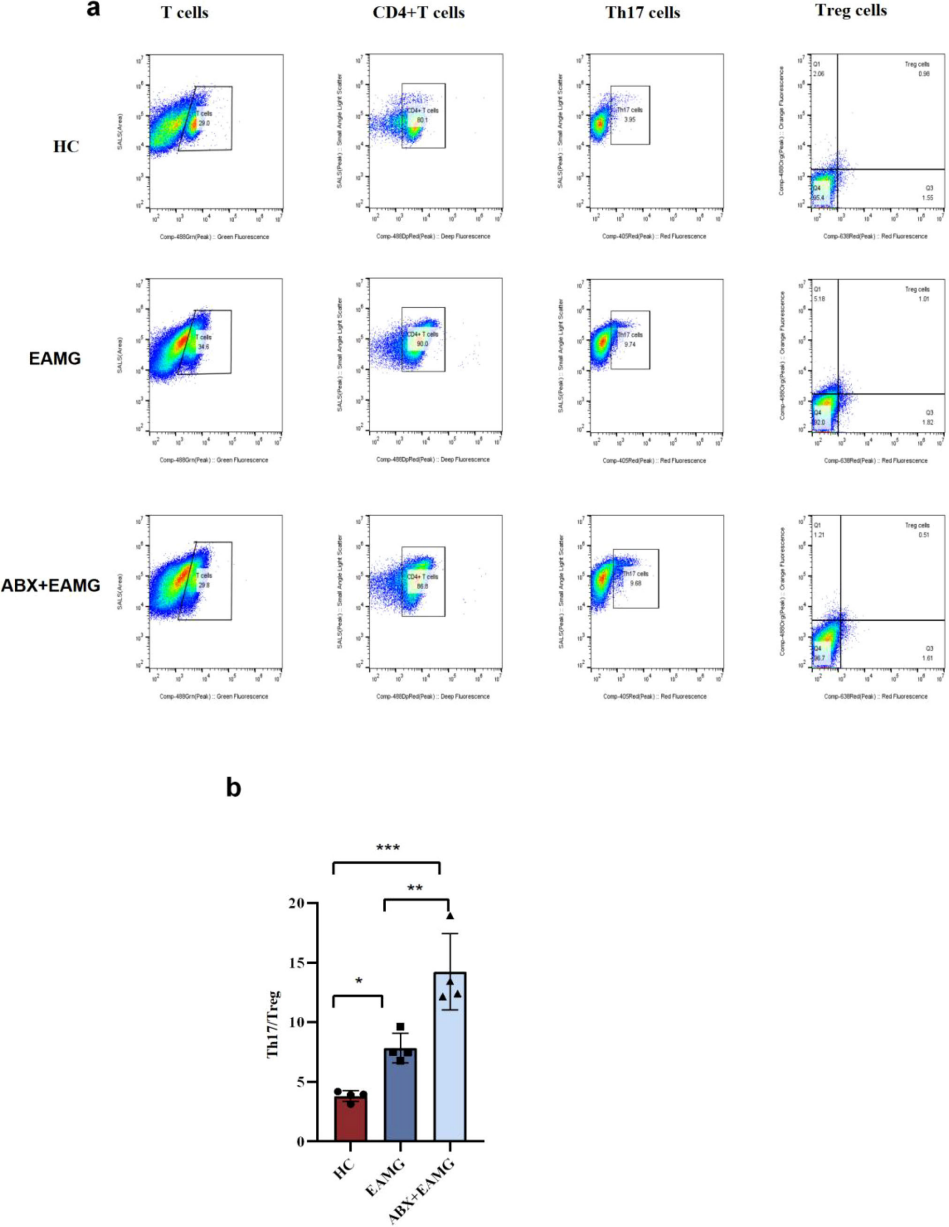
**(a)** Analysis of Th17 cells and Treg cells by flow cytometry in spleen. Spleen Th17 cells and Treg cells expression in HC, EAMG, ABX+EAMG groups. The Th17 cells were represented by CD3+CD4+CD25+IL-17A. CD3+CD4+CD25+Foxp3+ represents Treg cells. **(b)** The proportion of Th17/Treg cells in the three groups was compared. We found that the proportion of Th17/Treg cells in the EAMG group was significantly higher than that in the HC group and lower than that in the ABX+EAMG group, and the difference was statistically significant. Data was expressed as mean ± SD (n=4). (ns, no statistical significance; *p < 0.05, **p < 0.01; ***p < 0.001, ****p <0.0001).

Gut microbiota dysbiosis can promote an imbalance between Th17 and Treg cells. Th17 and Treg cells maintain immune homeostasis through mutual antagonism within the cellular immune system. We quantified splenic Th17 (CD3+CD4+IL-17A+) and Treg (CD3+CD4+CD25+Foxp3+) cells by flow cytometry on Day 72. Data were analyzed using FlowJo version 10.8.1 (**Figure 11a**). The Th17/Treg ratio in the ABX+EAMG group was significantly higher than those in both the HC (*p* < 0.001) and EAMG groups (*p* < 0.01) (**Figure 11b**). These findings suggest that gut microbiota dysbiosis compromises intestinal barrier function, which may lead to splenic Th17/Treg imbalance and elevated serum AChR-Ab levels. However, the specific mechanism underlying these alterations requires further in-depth study.

## 4 Discussion

It has been established that the gut microbiota of EAMG exhibits distinct characteristics. Altered gut microbiota can modulate serum AChR-Ab levels via fecal microbiota transplantation (FMT), suggesting a bidirectional interaction between the gut microbiota and EAMG. In addition, we further demonstrated that gut microbiota dysbiosis exacerbates the systemic inflammatory response in EAMG, impairs intestinal barrier function, and worsens the Th17/Treg cells immune imbalance. However, the sample size of this study is relatively limited. The statistical power of some analyses may be constrained by the sample size. In the future, larger-scale validation cohorts should be conducted to further explore the specific molecular mechanisms by which microbiota dysbiosis leads to the progression of EAMG and potential therapeutic directions.

The composition of the gut microbiota is closely related to intestinal barrier function. The structural integrity of intestinal tissues plays a vital role in maintaining barrier function. In our experiment, HE staining and immunofluorescence analysis of the colon were used to assess intestinal barrier function. We observed that the colon of EAMG and Lewis rats transplanted with EAMG-associated gut microbiota exhibit reduced Claudin-1 and Muc2 expression. Moreover, microbiota dysbiosis can exacerbate the intestinal barrier impairment in EAMG. Antigen presenting cells (APCs) and the intestinal microbiota jointly protect the body against infection, which can maintain the immune tolerance of the normal intestinal microbiota in the intestine, thereby maintaining the immune homeostasis of the host. Intestinal Toll-like receptor signaling plays an important role in maintaining enteric immune homeostasis (Kawai and Akira, 2010). When the structure of the intestinal microbiota undergoes drastic changes, causing severe damage to the intestinal barrier function, the TLR signaling will be overactivated, triggering a severe immune response (Abreu, 2010).This procedure can lead to disorder expression of inflammatory cytokines. The systemic immune response involves a mutual regulatory effect between immune molecules and immune cells, which is particularly important in autoimmune diseases.

B cells and T cells are both important components of the acquired immune system. AChR-Ab is a characteristic autoantibody of MG, which is secreted by B cells. The function of B cells is of great significance in MG, not only in individuals with positive AChR-Ab. In MG patients with negative acetylcholine receptor antibodies, overexpression of B cell marker genes was also observed in the thymus (Le Panse et al., 2006). Therefore, B cells are of great significance in the pathological mechanism of MG. CD4^+^ T lymphocytes are the main driving factor of MG. Th1, Th17 and Tregs all originate from CD4^+^ T lymphocytes. Previous studies have shown that Th17/Treg cell imbalance contributes to MG progression (Chen and Tang, 2021; Lu et al., 2023).

The gut microbiota can affect the Th17/Treg balance through multiple pathways, including influencing the production of gut microbiota metabolites, altering the development and function of immune cells, regulating cytokine secretion, and affecting inflammatory responses. The most common categories of gut microbiota metabolites include short-chain fatty acids, bile acid derivatives, amino acid metabolites, etc. The 3-phenylpropionic acid was found to enhance the intestinal epithelial barrier by activating the AhR signaling pathway, which can promote the differentiation of Treg and inhibit the generation of Th17 cells (Hu et al., 2023). SCFAs (Short-chain fatty acids) may be involved in the regulatory mechanisms of T cells and B cells. In obesity, butyrate can enhance the function of Treg cells and indirectly suppress the excessive activation of B cells (Zhi et al., 2019). The gut contains a large number of immune cells, including B cells and T cells in gut-associated lymphoid tissue (GALT), which are regulated by the gut microbiota to recognize and respond to pathogens and harmful substances (Xie et al., 2025). Intraepithelial T lymphocytes can regulate GLP-1 (Glucagon-Like Peptide-1) expression, thereby affecting inflammation and metabolic responses (He et al., 2019).The gut microbiota is closely related to intestinal mucosal immunity. The research of Marques transplanted a specific pathogen-free gut microbiota into germ-free mice and observed an increase in the number of Th17 cells and Treg cells in the ileum and colon (Marques de Souza et al., 2025). It was also found that the gut microbiota of T cell-deficient mice changed, confirming the bidirectional regulatory effect of the immune system on the gut microbiota (Marques de Souza et al., 2025). The increase of the pro-inflammatory cytokine IL-6 is positively correlated with the differentiation of Th17 cells, while the up-regulation of the anti-inflammatory cytokine TGF-β can enhance the function of Treg cells in the experimental autoimmune encephalomyelitis model (Miyake et al., 2024). The cytokines IL-17 and IL-21, secreted by Th17 cells, can directly stimulate the proliferation and differentiation of B cells (Nurieva et al., 2007).

Given the antibody-mediated characteristics of MG, future studies should include a comprehensive assessment of B cell immunity to more fully reveal the mechanisms of microbiota-immune interactions. In recent years, research on the correlation of regulatory B cells in autoimmune diseases has also gradually increased. Studies have found that the IL-10 produced by Tregs also promotes the generation of regulatory B cells (Bregs) (Hu et al., 2025). Currently, there are relatively few studies on Bregs related to gut microbiota. In SLE, researchers have found that administering vancomycin treatment can increase the expression of Bregs, thereby inhibiting disease progression in lupus mice (Mu et al., 2020). In MG, the proportion of Bregs in peripheral blood decreases and their functions are impaired (Sánchez-Tejerina et al., 2022). Our research did not directly elucidate the detailed pathways by which the intestinal microbiota affects immune cells and molecules, but it provided a solid foundation for understanding the immune mechanisms underlying MG. In conclusion, the gut microbiota directly or indirectly regulates the activity of B cells and affects the expression of AChR-Ab. Subsequent research will further analyze the changes in multiple immune axes, such as T cells and B cells, to thoroughly elucidate how gut microbiota regulates humoral immunity in EAMG. This will provide a more accurate perspective for mechanism research.

Our research found that the expression levels of IL-17 and TNF-α in Lewis rats were positively correlated with the expression of AChR-Ab. Therefore, we speculate that the imbalanced immune inflammatory environment promotes the activity of autoreactive B cells and the production of high-affinity antibodies, leading to an increase in the serum AChR-Ab level in Lewis rats. Meanwhile, the molecular mimicry function of bacteria may also be involved in this process. Molecular mimicry is believed to initiate or exacerbate autoimmune responses by matching the sequence or structure of self-antigens (Cusick et al., 2012). Herpes simplex virus can undergo an immune chemical reaction with the acetylcholine receptor alpha-subunit, and this cross-immune reaction may be related to certain cases of myasthenia (Schwimmbeck et al., 1989). Additionally, the autoantigenic site of the acetylcholine receptor alpha-subunit has 50% homology with the peptide segment of Haemophilus influenzae, and this peptide has been proven to have protective effects against experimental autoimmune myasthenia gravis (EAMG) and can weaken the induction and progression of EAMG (Im et al., 2002). Lewis rats with the EAMG intestinal microbiota (MMb) have significantly higher serum LPS expression levels than those with the HC intestinal microbiota (HMb). Rats in the MMb group showed more severe intestinal barrier damage. This indicates that the intestinal barrier damage caused by the imbalance of the microbiota may provide conditions for bacterial translocation. We speculate that intestinal bacterial translocation may also induce the immune response through a similar mechanism, leading to an increase in AChR-Ab expression levels. In the future, it may be possible to screen for the presence of antibodies against specific “mimetic” bacterial antigens in MG to assist in diagnosis or disease classification.

Splenic Th17/Treg cells serve as key indicators for evaluating systemic inflammation induced by gut microbiota alterations (Zhang et al., 2021). Our study not only identified Th17/Treg cell imbalance in EAMG but also demonstrated its crucial role in disease pathogenesis. Furthermore, we demonstrated that microbiota dysbiosis promotes EAMG progression by exacerbating Th17/Treg cell imbalance. However, the specific molecular mechanism still requires further investigation. Metabolites related to the gut microbiota can also establish a connection with autoimmune diseases by acting on the Th17/Treg balance. In EAMG, it was found that the restoration of butyrate (a short-chain fatty acid) can regulate the Th17/Treg balance in the spleen and lymph nodes (Smith et al., 2013).

Based on our experimental results, we hypothesize that there might be a vicious cycle in EAMG: the immune disturbance caused by the AChRα peptide may trigger the disease. Once the disease occurs, the activated immune cells and inflammatory environment further exacerbate the imbalance of the microbiota and the immune system. Moreover, the imbalance of the microbiota may intensify the autoimmune response by altering the related metabolites of intestinal microorganisms, changing intestinal permeability, bacterial translocation, and directly or indirectly affecting the immune cell processes. In Graves’ disease, dysbiosis can promote the dysregulated balance of Th17 to Treg cells, thereby enhancing the autoreactivity of B cells (Hou et al., 2021). Our findings establish a therapeutic strategy targeting intestinal microbiota restoration and Th17/Treg cell rebalancing for MG treatment innovation. Our research employed 16S rRNA gene sequencing, which could only reveal the composition of the intestinal microbiota. The heterogeneity of gut microbiota is attributed to various factors, including genetic factors, age, diet as well as different stages of the disease, varying severity and diverse clinical manifestations. All of these factors can shape unique microbial community structures. In different research groups, specific microbial species associated with the disease may vary. The composition of the gut microbiota shows significant heterogeneity among individuals, which is a fundamental characteristic in this research field. The connection between microorganisms and diseases is related not only to the species composition but also to their interactions with the host. Different microbial species may have similar biological functions. In MG, there are also changes in the related metabolites of intestinal microorganisms. Qiu et al. found that the content of short-chain fatty acids (SCFA) in the feces of MG patients decreased (Qiu et al., 2018). Ding et al. found that the expression of oleic acid metabolites in the intestines of MG patients increased (Ding et al., 2023).

Probiotics have shown therapeutic potential in some intestinal-related and immune-related diseases (Ahmadi et al., 2025; Meng et al., 2023; Sun et al., 2025). Moreover, in studies of EAMG and experimental encephalomyelitis, *Bifidobacteria* and *Lactobacilli* were found to have therapeutic potential (Consonni et al., 2018). Building on these findings, our research observed that the relative abundances of *Lactobacillus johnsonii* and *Bifidobacterium globosum* showed consistent changes before and after fecal microbiota transplantation. We speculate that *Lactobacillus johnsonii* and *Bifidobacterium globosum* have therapeutic potential for EAMG based on their abundance patterns. However, whether these species have therapeutic value for myasthenia gravis requires further investigation. Apart from the probiotics, the metabolites produced by gut microorganisms also show potential in treating diseases by improving the intestinal barrier. However, the reduction of SCFAs caused by dysbiosis may weaken this effect, thereby exacerbating the autoimmune response (Kovatcheva-Datchary and Arora, 2013). In MG, butyrate has been found to restore the function of Treg cells through the mTOR pathway (He et al., 2024). In the future, multi-omics techniques, such as metagenomics and metabolomics, should be combined to more comprehensively explore the association between the microbiota and MG.

## Data Availability

The datasets presented in this study can be found in online repositories. The names of the repository/repositories and accession number(s) can be found below: https://www.ncbi.nlm.nih.gov/, PRJNA1249227.
