# Supplementary figures and images for: Disruption of intestinal barrier and immune homeostasis links gut microbiota dysbiosis to aggravated experimental autoimmune myasthenia gravis
Source: Front Cell Infect Microbiol. 2026 Apr 10;16:1726788. doi: 10.3389/fcimb.2026.1726788 (PMC13106172; doi:10.3389/fcimb.2026.1726788)

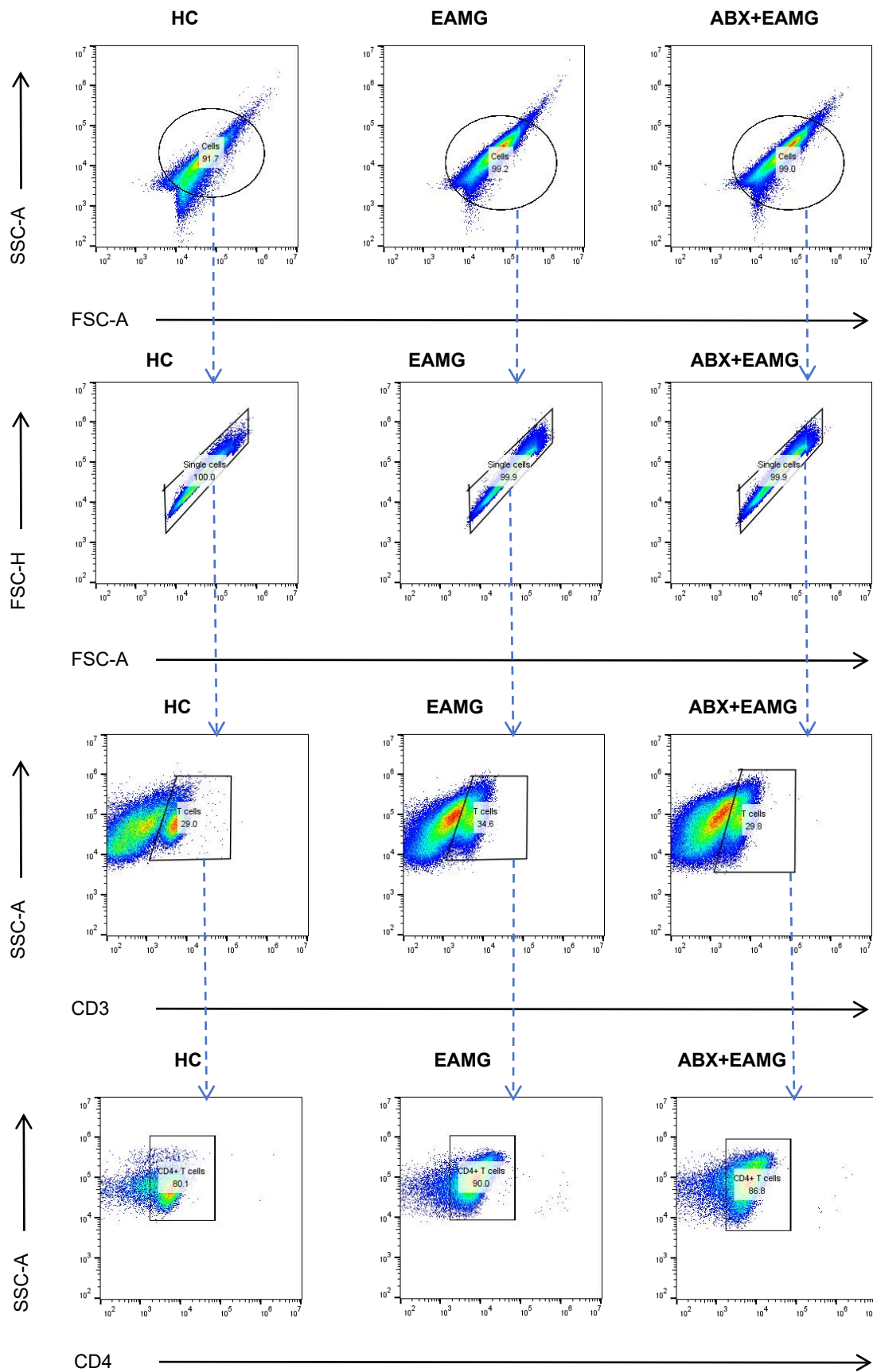

Supplemental Figure 1

Gated on CD4<sup>+</sup>T cells

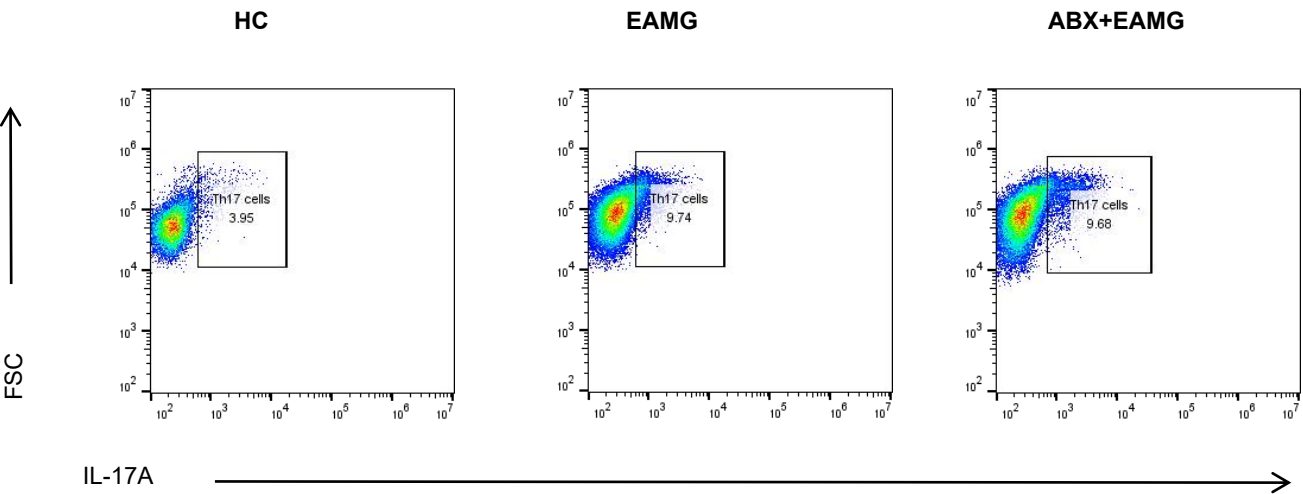

Gated on CD4<sup>+</sup>T cells

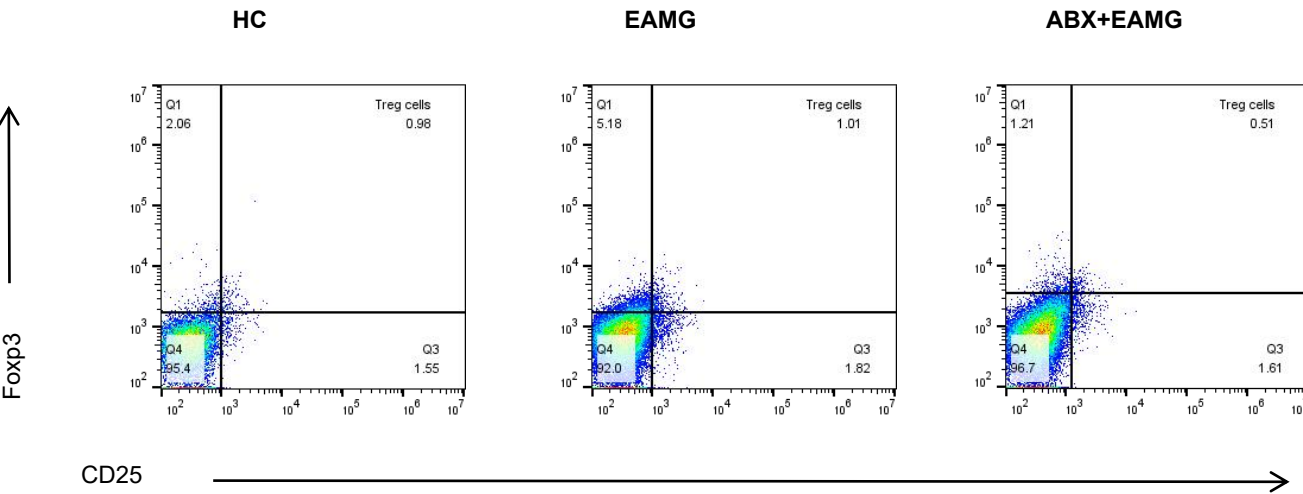

Supplemental Figure 2

Supplement: Supplementary file 1 [file Supplementaryfile1.pdf]
