# Supplementary material for: Disruption of intestinal barrier and immune homeostasis links gut microbiota dysbiosis to aggravated experimental autoimmune myasthenia gravis
Source: Front Cell Infect Microbiol. 2026 Apr 10;16:1726788. doi: 10.3389/fcimb.2026.1726788 (PMC13106172; doi:10.3389/fcimb.2026.1726788)
Supplement: Supplementary file 2 [file Supplementaryfile2.docx]

**Supplementary Figure legends**

Supplementary Figure 1. The figure presents the gating stategy. Firstly, the FSC-A/SSC-A operation is carried out to screen the cell population. Then, the FSC-H/FSC-A operation is conducted to eliminate duplicate cells. Next, CD3 staining is performed to identify the T cell population under study. Finally, CD4 staining is carried out to determine the CD4+ T cells under investigation.

Supplementary Figure 2. The figure presents the proportions of IL-17A cell staining and CD25+Foxp3 staining cells in the HC group, EAMG group, and ABX+EAMG group based on the CD4+ T cell gating channel.
